# Ecological risk assessment of aquatic organisms induced by heavy metals in the estuarine waters of the Pearl River

**DOI:** 10.1038/s41598-023-35798-x

**Published:** 2023-06-05

**Authors:** Zhihua Tang, Xinyu Liu, Xiaojun Niu, Hua Yin, Minru Liu, Dongqing Zhang, Huafang Guo

**Affiliations:** 1 Integrated Technology Center, Guangzhou Institute of Energy Conversion, Chinese Academy of Sciences, Guangzhou, 510640 China; 2 Bureau of Hydrology and Water Resources, Pearl River Water Resources Commission of Ministry of Water Resources, Guangzhou, 510611 China; 3 School of Environment and Energy, South China University of Technology, Guangzhou, 510006 China; 4 Guangdong Provincial Key Laboratory of Petrochemical Pollution Processes and Control, School of Environmental Science and Engineering, Guangdong University of Petrochemical Technology, Maoming, 525000 China

**Keywords:** Environmental sciences, Ocean sciences, Risk factors

## Abstract

With the rapid economic development of China's coastal areas and the growth of industry and population, the problem of heavy metal contamination in estuarine waters is increasing in sensitivity and seriousness. In order to accurately and quantitatively describe the current status of heavy metal contamination and identify sensitive aquatic organisms with high ecological risks, five heavy metals in eight estuaries of the Pearl River were monitored at monthly intervals from January to December in 2020, and the ecological risks of aquatic organisms induced by heavy metals were evaluated using Risk quotients (RQ) and species sensitivity distributions (SSD) methods. The results showed that the concentrations of As, Cu, Pb, Hg and Zn in estuaries of the Pearl River were (0.65–9.25) μg/L, (0.07–11.57) μg/L, (0.05–9.09) μg/L, (< 0.40) μg/L and (0.67–86.12) μg/L, respectively. With the exception of Hg in Jiaomen water, the other heavy metals in each sampling site met or exceed the water quality standard of Grade II. The aquatic ecological risks of As, Pb and Hg were generally low in the waters of the Pearl River estuary, but individual aquatic organisms are subject to elevated ecological risks due to Cu and Zn. The content of Zn has a lethal effect on the crustaceans *Temora Stylifera*, and the content of Cu has a serious impact on the mollusks *Corbicula Fluminea* and has a certain impact on the crustaceans *Corophium sp.* and the fish *Sparus aurata*. Heavy metal levels and joint ecological risks (msPAF) in the Humen, Jiaomen, Hongqimen, and Hengmen estuaries were slightly higher than in other estuaries, and the Yamen estuary had the lowest contration of heavy metals and ecological risk. Research findings can serve as a basis for formulating water quality standards for heavy metals and for protecting aquatic biodiversity in the Pearl River Estuary.

## Introduction

With the rapid economic development of China's coastal areas and the growth of industry and population, the impact of anthropogenic activities on the environment of coastal waters is becoming increasingly important, and heavy metal contamination in estuaries is becoming increasingly significant and serious^1–5^. Heavy metals that discharge into the ocean are derived from natural and anthropogenic sources. Natural sources, including crustal rock weathering, underwater volcanic eruption, and water loss and soil erosion on the continent, are the bottom values for marine heavy metals. The anthropogenic sources are mainly industrial and mining wastewater discharges, pesticides losses, and fossil fuel combustion emissions. Of these, river discharges are the principal direct source of toxic heavy metals to coastal waters^6^. In 2017, heavy metal discharges from the Pear River into the South China Sea were reported to be 3500 tons, containing 3000 tons of (Cu, Zn, Cd, Pb and Hg), and 500 tons of As^7^.

These toxic heavy metals threaten the health and survival of aquatic organisms by virtue of their high toxicity, bioaccumulation and persistence^8^. Heavy metal contamination has been detected in several nearshore waters in China, and elevated heavy metal concentrations in fish, shrimp, crab, and mollusk species have been observed in many adjacent marine areas^3,5,9–19^. When heavy metals enter into the organism, they tend to combine with enzyme proteins, destroy enzyme activity, affect the normal physiological activities of the body, and cause function abnormality of nervous, respiratory, digestive and excretory systems, leading to chronic poisoning and even death^20–23^. As a result of genotoxic damage caused by heavy metals, sperm motility in aquatic animals will be decreased, affecting reproduction and biodiversity^24–26^. Certain heavy metals will be transformed into more toxic organic compounds after their introduction into organisms, and the biological toxicity will be further amplified. For example, Hg can be converted to more toxic methylmercury after being ingested by microorganisms in water^6,27^. After these microorganisms have been eaten by fish, prawns or other aquatic animals, methylmercury enters into the human body through the food chain and threatens human health. When the dose of heavy metals in the human body accumulates at a certain level, it causes non-carcinogenic diseases or a carcinogenic risk^28^.

Ecological risk assessment of aquatic organism is a quantitative assessment technology developed in 1970s, which is to evaluate the possibility, proportion, and the extent of the potential adverse effects on aquatic communities when aquatic organisms are exposed to one or several pollutants stressors^29^. The weight of evidence (WOE) plays an important role in assessing aquatic biological risks by integrating environmental assessment information and the chain of evidence^30^. The commonly used evidence chain is chemical analysis, biological toxicity test and ecological investigation. The US Environmental Protection Agency (US EPA) collects extensive data on aquatic toxicity for different heavy metal species, concentrations, and toxicity effect endpoints, and maintains an online database (ECOTOX database) for researchers to explore^31^. Most toxicity tests were carried out in the laboratory, and the EC50 (median effect concentration) and LC50 (median lethal concentration) are the most commonly used toxicity effect endpoints for ecological assessment of aquatic organism^10,32^. The interspecies correlation estimation (ICE) models and a species sensitivity distributions (SSD) as scientific methods to predict and evaluate the hazards of single heavy metal and synergistic toxicity of multiple heavy metals have been widely used in the risk assessment of aquatic ecosystem since 1990s^33^. The Causal Analysis/Diagnosis Decision Information System (CADDIS) provides detailed guidance for the generation of SSD, including selection of toxicological data, environmental adaptation judgment and identification of stress factors etc.^34^. Zheng et al. applied SSD method to assess acute the toxic effect of aquatic organisms induced by six heavy metals (including Cu, Hg, Cd, Cr ^6+^, Pb and Zn), and the results indicated that invertebrate taxa exhibited higher sensitivity than vertebrates for each heavy metal^35^. Wang et al. constructed SSD curves to evaluate ecological risks of marine organisms (including crustacean, fish and mollusc) induced by five heavy metals (including Cu, Zn, Pb, Hg and As) in China’s coastal waters, and found that there were a certain high ecological risk point in Bohai Sea caused by Cu and Zn^10^. Park and Kim applied the SSD method to obtain the ecological risk threshold values of Cd, Cu, Pb and Zn for aquatic organisms living in Korea^36^. When evaluating the risk of aquatic organisms induced by heavy metal, SSD and ICE take into account the relationship between different species, the uncertainty caused by model selection, data availability and variances, and obtain the prediction and confidence interval through probability statistics, which greatly improves the reliability and credibility of the assessment results^32^.

Over the past few years, many researchers have studied heavy metal contamination in the Pearl River estuary, but most studies focus on heavy metals in sediments^3,37^, and there are few studies on the ecological risks of local aquatic species induced by heavy metals in water. In this study, we monitored five heavy metals (including As, Cu, Pb, Zn and Hg) in eight estuaries of the Pearl River for twelve consecutive months, collected and sorted out ecotoxicity data of aquatic organisms from the US EPA ECOTOX database, developed SSD curves and adopt an ICE model to carry out ecological risk assessment. It is hypothesized that the effects of heavy metals present in sediments and other pollutants found in water on aquatic organisms have yet to be taken into account. The purpose of this study is to accurately and quantitatively describe the current status of heavy metal contamination in estuarine waters of the Pearl River, identify sensitive aquatic species with high ecological risk, and provide a decision-making basis for ecological risk control and ecological modification. The innovation of this study lies in the comprehensive utilization of monthly hydrological monitoring data and risk assessment models to monitor water quality in the Pearl River Estuary, enabling rapid identification of aquatic species facing ecological risks.

## Methods and materials

### Sample collection and heavy metal testing

The Pearl River is comprised of the Xijiang, Beijiang, Dongjiang and the Pearl River Delta rivers. The mainstream Xijiang River originates from the eastern foot of the Maxiong Mountain in the vein of the Wumeng Mountain in Qujing City, Yunnan Province, and flows from west to east to Sixian Channel in Sanshui City, Guangdong Province, and flows into the Pearl River Delta. The Pearl River Delta flows into the South China Sea through Humen, Jiaomen, Hongqimen, Hengmen, Modaomen, Jitimen, Hutiaomen and Yamen, after the inflow of Xijiang, Beijiang, Dongjiang and other small and medium-sized rivers. The length of the main stream is 2214 km, with an average slope of 0.446‰. The total area of the basin is 453,700 km^2^, of which the domestic area is 442,500 km^2^. The annual average runoff of the Pearl River Basin is 338.1 billion m^3^, second only to the Yangtze River basin and ranking second among the seven major river basins in China.

**Figure 1:**
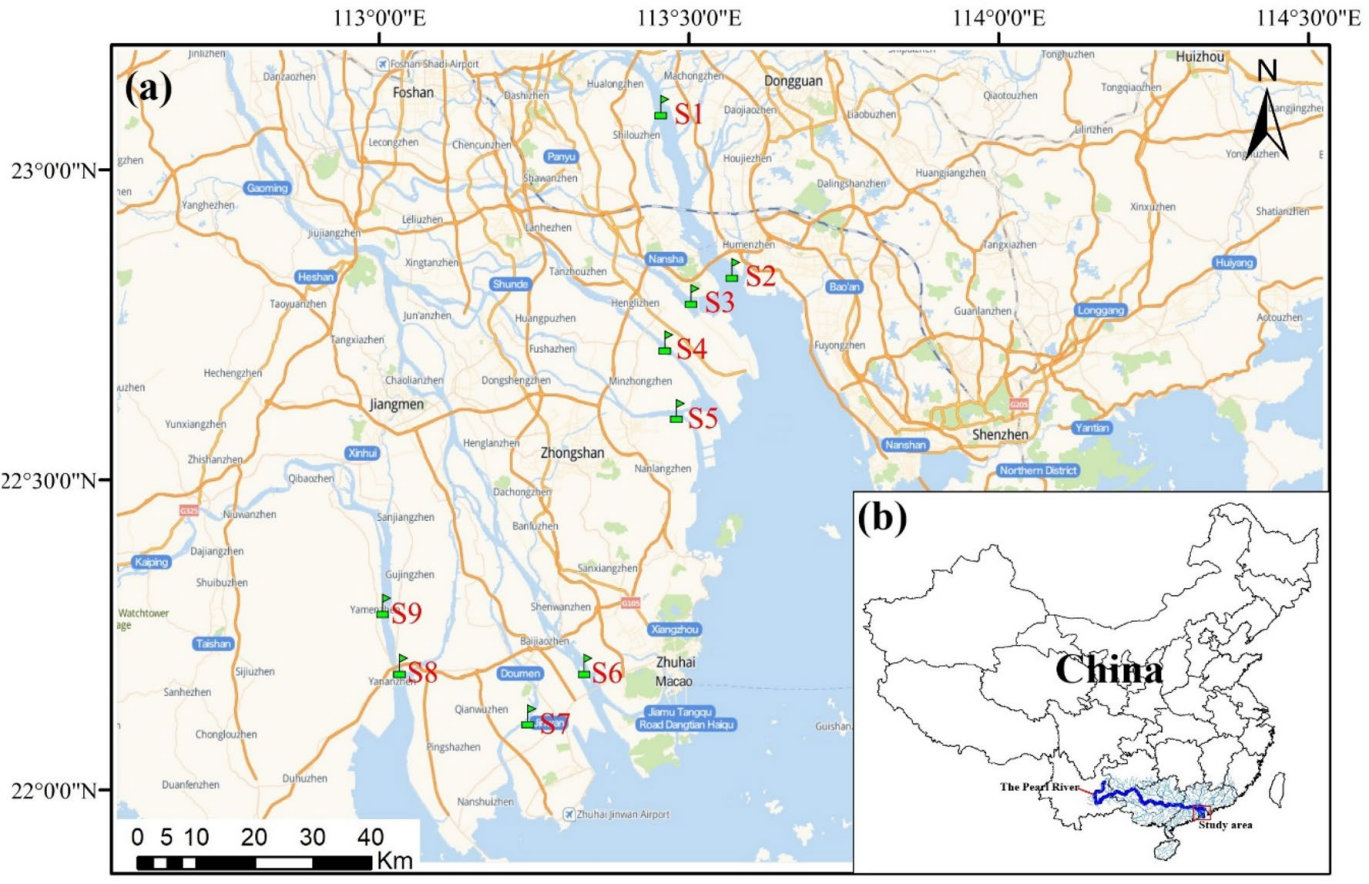
Distribution of rivers and sampling sites in the Pearl River estuary. **(a)** Nine sampling sites distributed in eight estuaries of the Pearl River; **(b)** Location of the Pearl River estuary in China. ArcGIS 10.2 software (www.arcgis.com) was used to generate this figure and the base map was come from Amap.

According to the guidance of “Specifications on Spot Location of Monitoring Sites Related to Coastal Area Environment (HJ730-2014)”, eight monitoring sections in rivers entering into the South China Sea are designed: S2 (Humen), S3 (Jiaomen), S4 (Hongqimen), S5 (Hengmen), S6 (Modaomen), S7 (Jitimen), S8 (Hutiaomen), and S9 (Yamen). Because of enormal volumes of water flow in the Humen estuary, according to the “Surface Water and Sewage Monitoring Technical Specifications (HJ-T91-2002)”, another river monitoring section S1 (Shijiaozui) was added to its upstream. Figure 1 shows the locations of sampling sites in the study area. Three sampling points were set at the left, middle and right of each monitoring section. Surface (~ 0.5 m below water surface), middle (half of the depth) and bottom (~ 0.5 m above the river bottom) water samples were collected at each sampling point, and then mixed evenly. Approximately 500 mL of the mixed water samples were filtered by 0.45 μm Millipore filter. Nitric acid (HNO_3_) as a guaranteed reagent was added to decrease the pH of the water less than 2 and the treated water samples were kept in an incubator at 4° C. Water samples were collected from the nine river channel monitoring sections at monthly intervals from January to December in 2020.

The As, Cu, Pb, Zn and Hg concentration in the water samples were tested using an Elan 6000 inductively coupled plasma mass spectrometer (Perkin Elmer, Waltham, MA, USA). The detection limits for As, Cu, Pb, Zn and Hg are 0.0006 μg/L, 0.0002 μg/L, 0.00004 μg/L, 0.0003 μg/L and 0.016 μg/L, respectively. If the measured values of heavy metal in water sample were below the detection limits, the concentration of heavy metals was assumed to be half of the detection limits^10^.

### Ecological risk assessment

The RQ method makes it possible to rapidly identify the types and risk areas of heavy metals posing ecological risks, which is widely used in the assessment of aquatic ecological risks^38^. RQ is the ratio of the concentration of heavy metals to their reference toxicity values, which is defined as follows:

1 $${\text{RQ}} = {\text{HMC}}/{\text{TOX}}$$

where HMC is the content of heavy metals in water (μg/L); TOX is the reference toxicity value, and HC_5_ (5% Hazardous Concentration) is generally used as the reference toxicity value^38^. Depending on the species sensitivity distribution, HC_5_ represents the concentration of heavy metals at a cumulative rate of 5% of the affected species, or concentrations that would protect 95% of the species^10,32,39^. When the QR < 0.10, the ecological risk of heavy metals on aquatic organisms is low. When 1.00 ≥ QR ≥ 0.10, heavy metals have a certain risk to the aquatic organisms, although it is not very serious, control or remedial measures should be taken as quickly as possible. When QR > 1.00, heavy metals pose a relatively high ecological risk to aquatic organisms, and it is necessary to take urgent action to control pollution^38^.

SSD describe the probability distribution of sensitivity of different species to a pollutant because of their life cycle, physiological structure, behavioural characteristics and geographical distribution. SSD curves can be bulit with laboratory toxicological data on target organisms. SSD curve establishes a correlation between pollutant concentration and the proportion of species affected, which could assist in the determination the proportion of species with adverse effects of a given pollutant concentration. As recommended by previous studies^10,32^, the EC_50_ or LC_50_ values from the US EPA ECOTOX database were adopted to develop SSD curves in this study. LC_50_ and EC_50_ represent the concentration at which half of the individuals exposed die and reproduction is reduced by 50%, respectively. According to the results of the oceanographic survey, there are mainly 113 species of fish, 23 species of crustaceans and 12 species of mollusks in the estuary of the Pearl River^40,41^. In this study, the EC_50_ and LC_50_ values of above 148 aquatic species (fish, crustaceans and mollusks) were searched in the US EPA ECOTOX database according to their Latin nomenclature and scientific name, and the principles of ecotoxicity data selection are as follows: (a) The exposure media were freshwater or saltwater and the test locations were laboratories or all field tests. (b) Forms of heavy metal exposed to aquatic species were inorganic, and organic forms of heavy metals were excluded during data selection because of the absence of test data. (c) At least 10 toxicity tests on aquatic species for each heavy metal are necessary to construct SSD curves and ICE models. The species sensitivity distribution generator recommended by the US EPA was chosen to perform the specific process of SSD curves construction and uncertainty calculation. The SSD generator applies the linearized log-normal distribution of data for concentrations at which different species exhibit a standard response to a stressor^42^.

Based on the SSD curves and heavy metal contents in the Pearl River estuary waters, the ecological risk and the potential affected fraction (PAF) of aquatic organisms induced by a single heavy metal at prescribed concentrations were evaluated. To describe the total ecological hazards from multiple substances, i.e., the PAF of aquatic organisms induced by the five heavy metals (msPAF), the following equation has been applied^10,43,44^:

2 $${\text{msPAF}} = {1} - \left( {{1} - {\text{PAF}}_{As} } \right) \times \left( {{1} - {\text{PAF}}_{Cu} } \right) \times \left( {{1} - {\text{PAF}}_{{{\text{Pb}}}} } \right) \times \left( {{1} - {\text{PAF}}_{Zn} } \right) \times \left( {{1} - {\text{PAF}}_{Hg} } \right)$$

where PAF_As_, PAF_Cu_, PAF_Pb_, PAF_Zn_ and PAF_Hg_ are the ecological risks of aquatic organisms under the exposure of As, Cu, Pb, Zn and Hg in the estuary of the Pearl River, respectively.

### Data treatment and statistics method

The ArcGIS 10.2 software was designed to display geographical data for study area and sampling sites. Statistical analysis was used to process the 12-month heavy metal data at each sampling site and figures were plotted using OriginPro 2020b. The descriptive statistical method was used to analyse the RQ values of each sampling site using IBM SPSS Statistics 20. Diagrams of ecological risks associated with heavy metals were also created using OriginPro 2020b. The SSD curves were created using SSD generator down from EPA’s website^42^.

### Research ethics

All authors have read, understood, and have complied as applicable with the statement on "Ethical responsibilities of Authors.

## Results

### Heavy metal concentrations in the estuarine waters of the Pearl River

**Figure 2:**
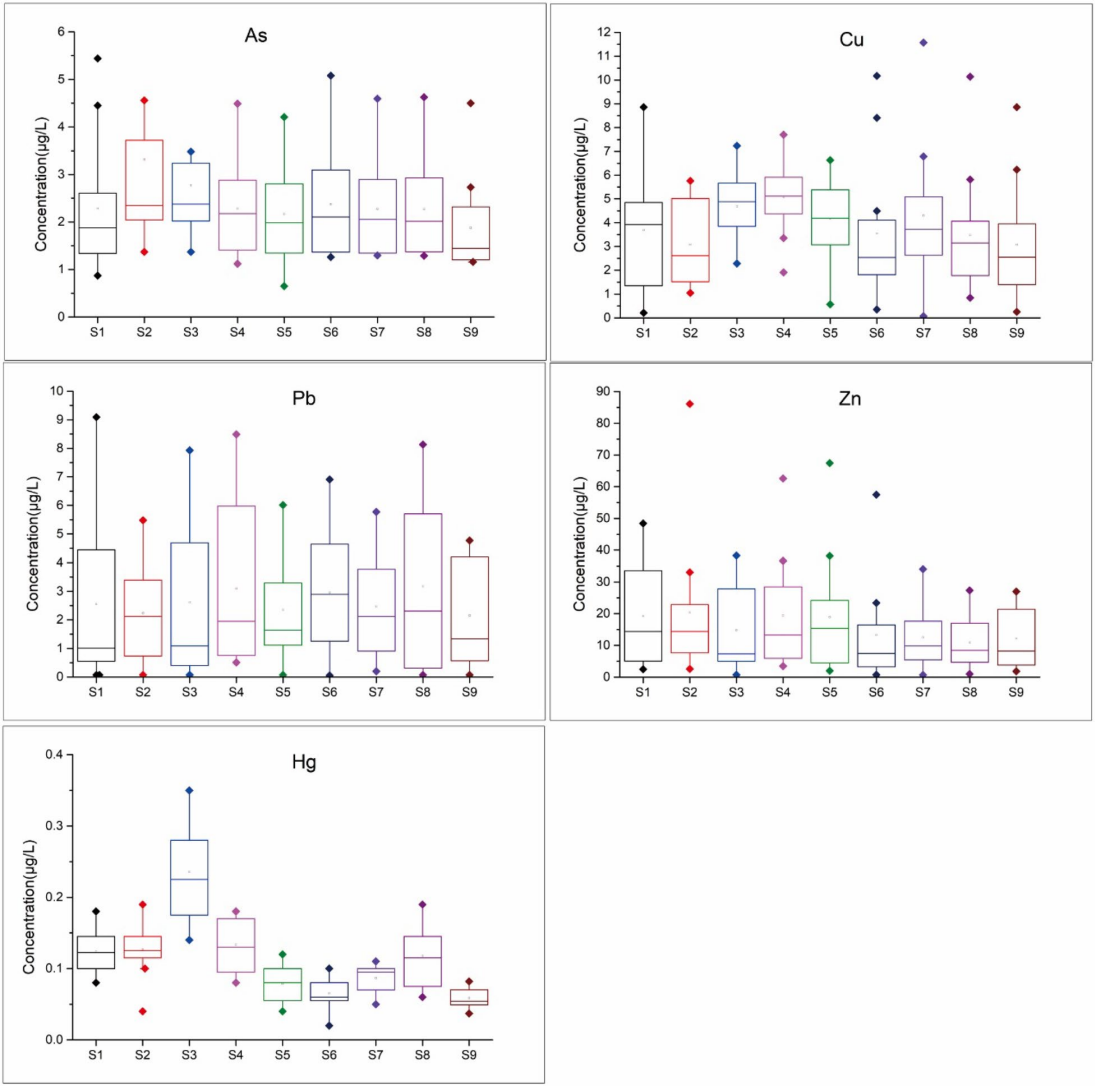
Concentrations of As, Cu, Pb, Zn and Hg in the estuarine waters of the Pearl River.

Statistics on heavy metal concentrations at 9 sampling sites distributed in estuaries of the Pearl River were illustrated in Fig. 2 as diagrams.

The result showed that the concentrations of As in the estuarine waters of the Pearl River were (0.65–9.25) μg/L with slightly higher average values in Humen (S2) and Jiaomen (S3) water than that of other sampling sites. The Cu concentrations in estuarine waters of the Pearl River were (0.07–11.57) μg/L, and the average concents of Cu in Humen (S2), Jiaomen (S3), and Hongqimen (S4) water were slightly higher than the other sampling sites. The Zn content in estuaries of the Pearl River ranged from 0.67 μg/L to 86.12 μg/L with slightly higher average values in Shijiaozui (S1), Humen (S2), Hongqimen (S4), and Hengmen (S5) water than those at other sampling sites. The highest average content of Pb occurred in Modaomen (S6) water with value of 3.17 μg/L. The concentration of Hg in the estuarine waters of the Pearl River was less than 0.40 μg/L, and the Jiaomen (S3) water had the highest average concentration.

### The species sensitivity distribution for As, Cu, Pb, Zn and Hg in estuarine waters of the Pearl River

**Figure 3:**
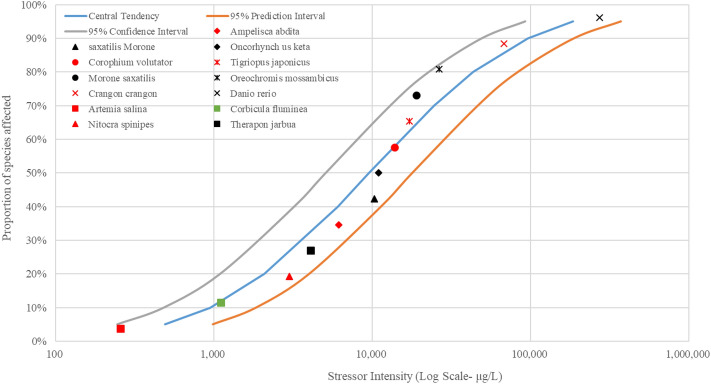
The SSD curves for As was developed using the US EPA ECOTOX database. Red, black, and green markings depict crustaceans, fish, and mollusks, respectively.

**Figure 4:**
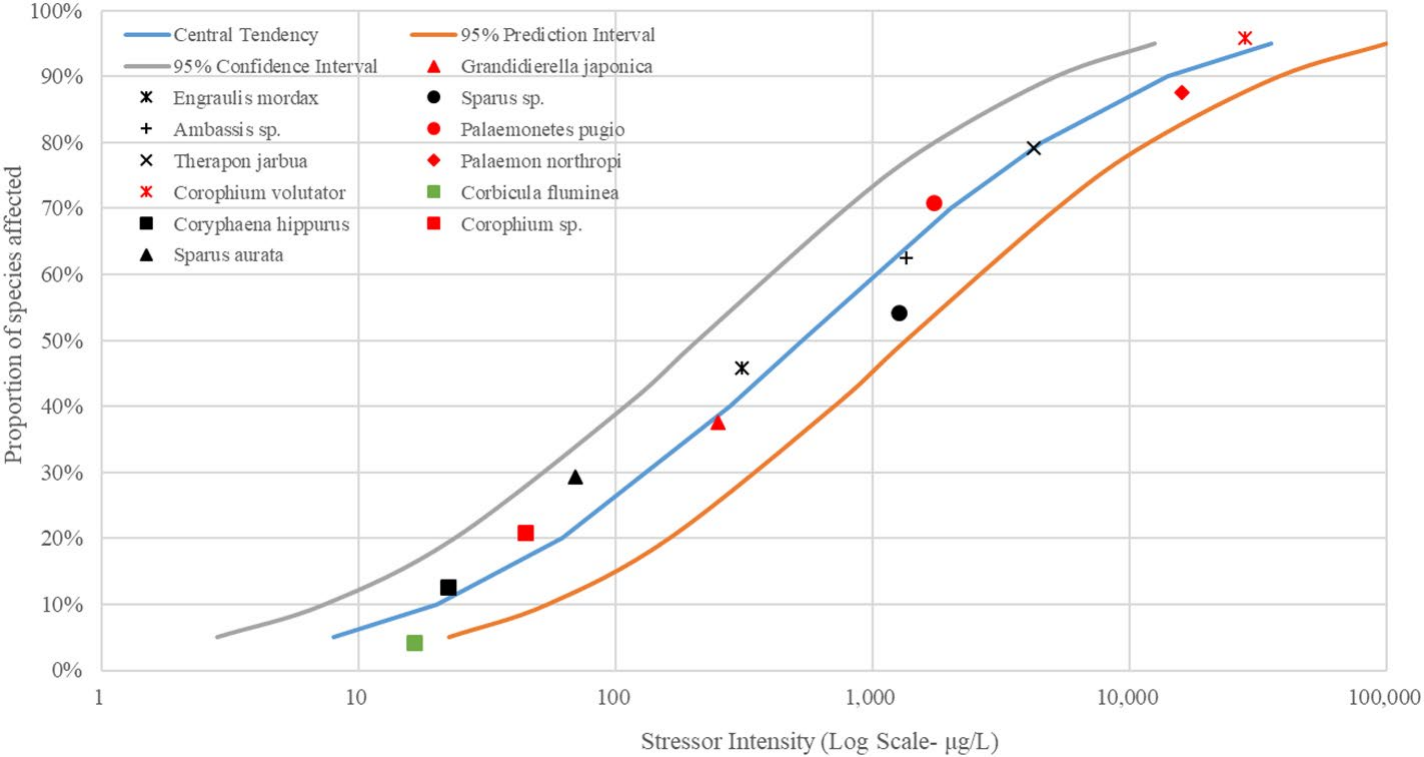
The SSD curves for Cu was developed using the US EPA ECOTOX database. Red, black, and green markings depict crustaceans, fish, and mollusks, respectively.

**Figure 5:**
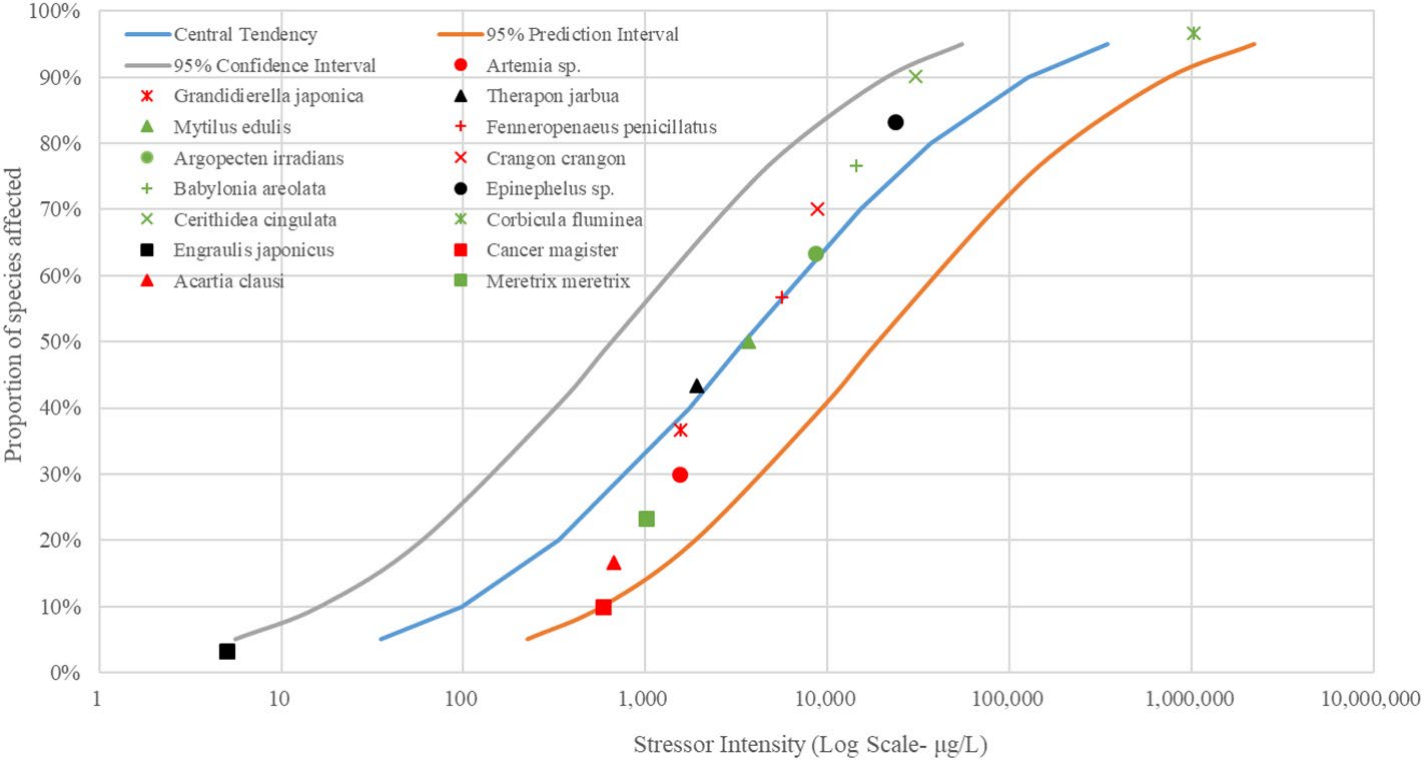
The SSD curves for Pb was developed using the US EPA ECOTOX database. Red, black, and green markings depict crustaceans, fish, and mollusks, respectively.

**Figure 6:**
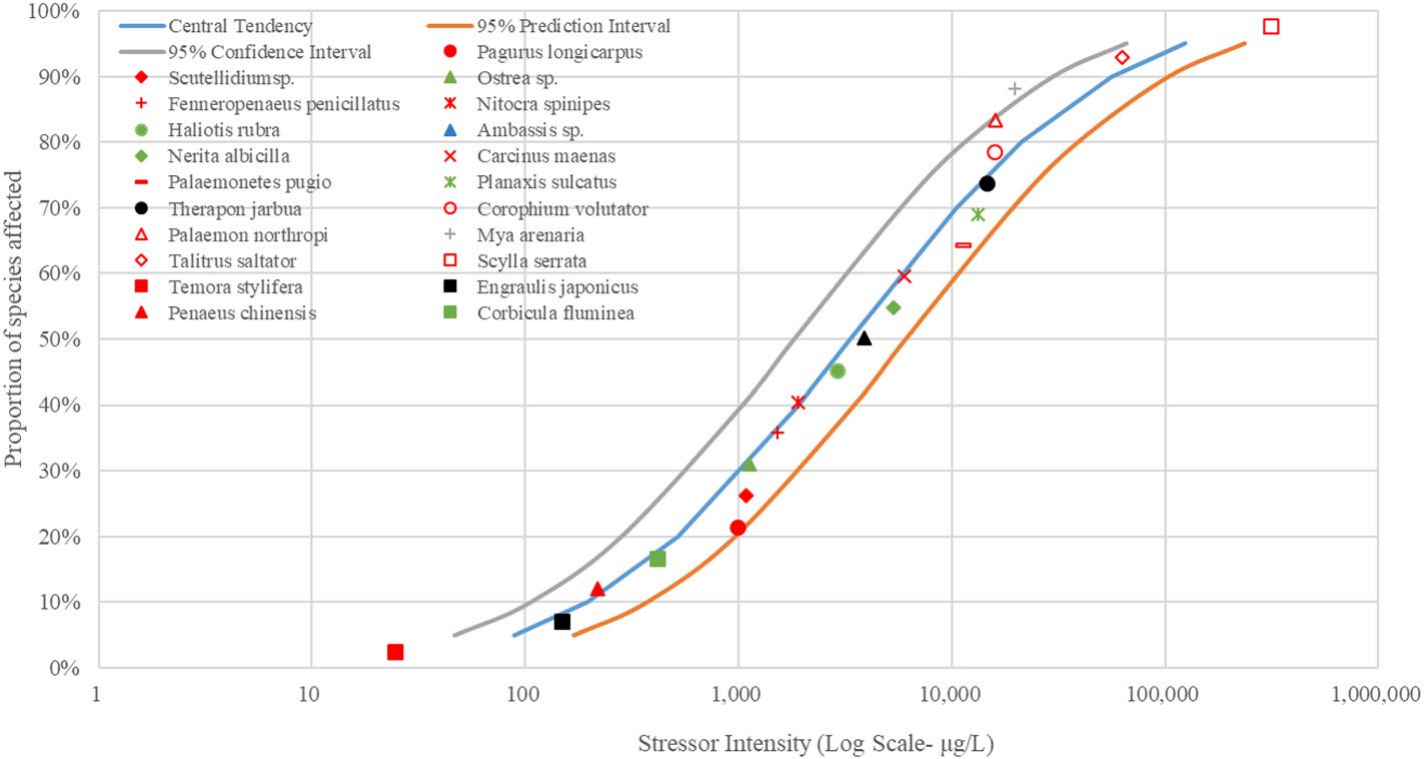
The SSD curves for Zn was developed using the US EPA ECOTOX database. Red, black, and green markings depict crustaceans, fish, and mollusks, respectively.

**Figure 7:**
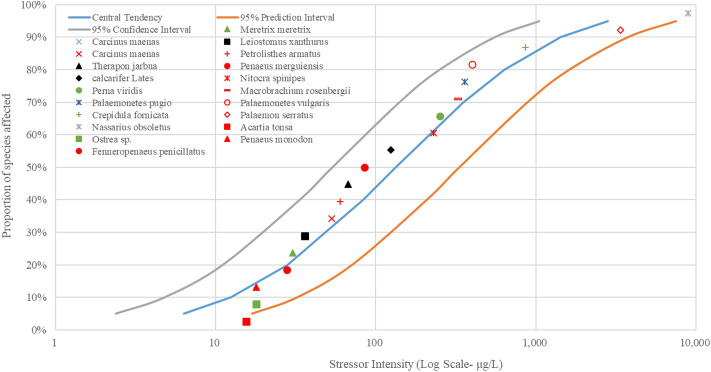
The SSD curves for Hg was developed using the US EPA ECOTOX database. Red, black, and green markings depict crustaceans, fish, and mollusks, respectively.

In this study, fish, crustaceans and mollusks in estuarine waters of the Pearl River were put together to generate the SSD curves due to the fact that only a few species’ EC_50_ or LC_50_ values were retrieved in the US EPA ECOTOX database. The SSD curves of As, Cu, Pb, Zn and Hg were illustrated in Figs. 3, 4, 5, 6, and 7, respectively.

The results of SSD curves showed that the proportion of species affected increased rapidly as the intensity of stressors (heavy metal concentrations in waters) increased. The crustacean *Artemia salina* showed the highest susceptibility to the heavy metal As, followed by the mollusk *Corbicula fluminea* (Fig. 3). The mollusk *Corbicula fluminea* was also the body most sensitive to heavy metal Cu (Fig. 4). The fish *Engraulis japonicus* showed the greatest sensitivity to heavy metal Pb (Fig. 5), and the crustacean *Temora stylifera* was the body most susceptible to heavy metal Zn (Fig. 6). For heavy metal Hg, the crustacean *Acartia tonsa* was the most sensitive organism, followed by the mollusk *Ostrea sp.* (Fig. 7). The marking position of aquatic organisms in the SSD curves was determined by individual differences, and there was no obvious difference in the distribution of species groups among crustacean, fish and mollusk species.

### Ecological risks of As, Cu, Pb, Zn and Hg in estuarine waters of the Pearl River

The HC_5_ values of As, Cu, Pb, Zn and Hg derived from the SSD curves and the RQ values of each heavy metal at all sampling sites calculated according Eq. (1) were listed in Table 1. The HC_5_ values for As, Cu, Pb, Zn and Hg to aquatic organisms were 490.59 μg/L, 7.97 μg/L, 35.43 μg/L, 88.78 μg/L and 6.35 μg/L, respectively. This means that if aquatic organisms are exposed to the same level of risk, heavy metal concentrations are ranked as follows: As > Zn > Pb > Cu > Hg. The mean values of RQ for As, Pb and Hg at all sampling sites were less than 0.10, suggesting that ecological risks of As, Pb and Hg on aquatic organisms were low in estuarine waters of the Pearl River. The mean values of RQ for Cu and Zn at all sampling sites were greater than 0.10 but less than 1.00, indicating that Cu and Zn have a certain risk to the aquatic organisms in estuarine waters of the Pearl River.

**Table 1:** The HC_5_ values (μg/L) derived from the SSD curves and the RQ values of each heavy metal at all sampling sites.

	As	Cu	Pb	Zn	Hg
HC_5_	490.59	7.97	35.43	88.78	6.35
RQ-S1	0.01 ± 0.00	0.46 ± 0.36	0.07 ± 0.08	0.22 ± 0.18	0.02 ± 0.01
RQ-S2	0.01 ± 0.01	0.39 ± 0.22	0.06 ± 0.05	0.23 ± 0.26	0.02 ± 0.01
RQ-S3	0.01 ± 0.00	0.59 ± 0.18	0.07 ± 0.08	0.17 ± 0.16	0.04 ± 0.01
RQ-S4	0.01 ± 0.00	0.68 ± 0.20	0.09 ± 0.08	0.22 ± 0.20	0.02 ± 0.01
RQ-S5	0.00 ± 0.00	0.52 ± 0.22	0.07 ± 0.06	0.21 ± 0.21	0.01 ± 0.00
RQ-S6	0.01 ± 0.00	0.44 ± 0.37	0.08 ± 0.06	0.15 ± 0.18	0.01 ± 0.00
RQ-S7	0.01 ± 0.00	0.54 ± 0.36	0.07 ± 0.05	0.14 ± 0.11	0.01 ± 0.00
RQ-S8	0.01 ± 0.00	0.44 ± 0.32	0.09 ± 0.09	0.12 ± 0.09	0.02 ± 0.01
RQ-S9	0.00 ± 0.00	0.39 ± 0.31	0.06 ± 0.05	0.14 ± 0.11	0.01 ± 0.00

**Figure 8:**
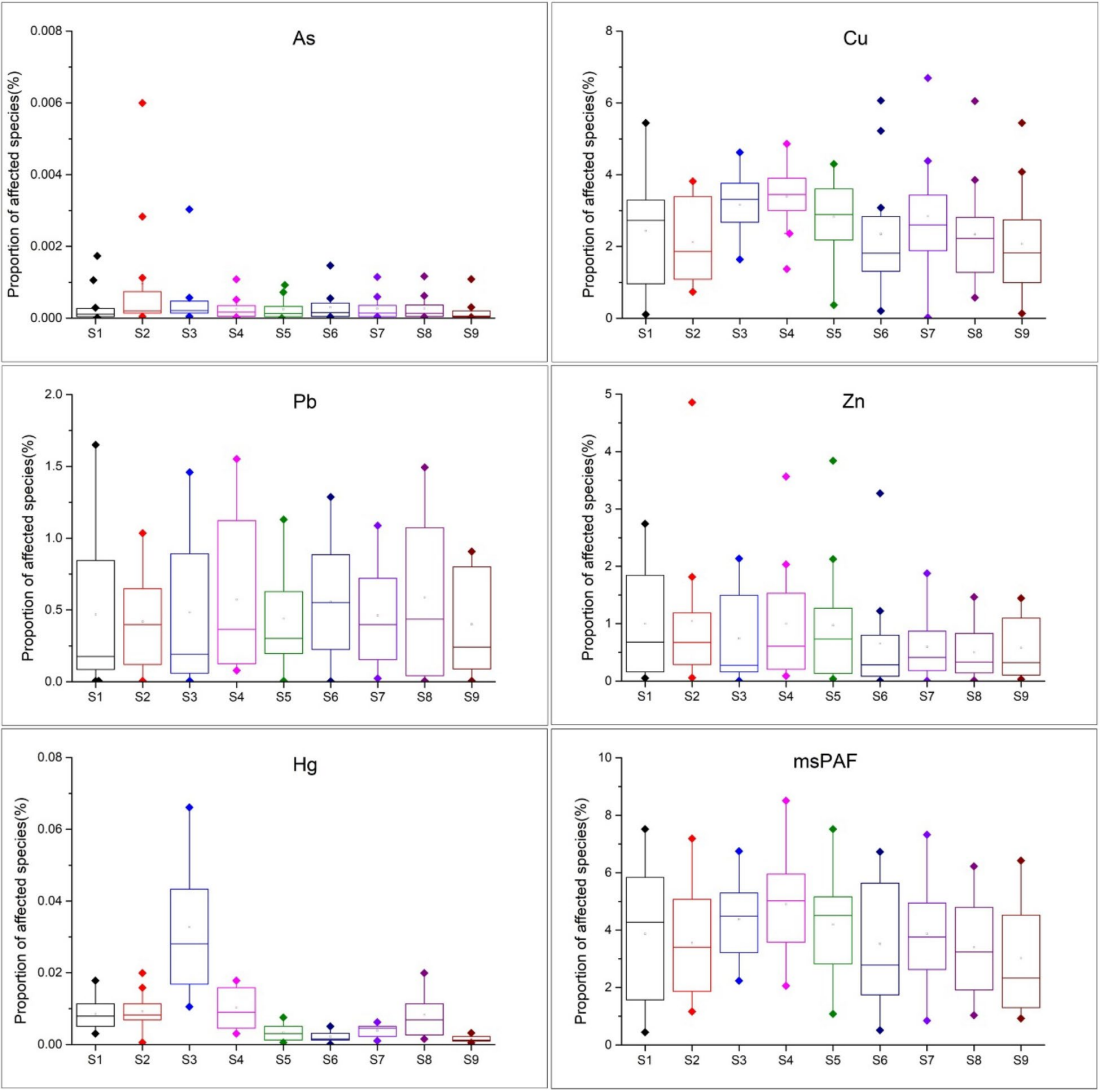
Ecological risks of the selected aquatic organisms induced by As, Cu, Pb, Zn and Hg.

Ecological risks faced by the aquatic organisms in estuarine waters of the Pearl River induced by single heavy metal and by the five heavy metals (msPAF) were illustrated in the Fig. 8. The results showed that the proportion of affected species induced by As and Hg were less than 0.008% and 0.08%, respectively. This means that risks related to aquatic organisms caused by As and Hg were low. The proportion of affected species caused by Cu, Pb, and Zn in estuarine wates of the Pearl River was (0.02–6.70%), (0.01–1.65%), and (0.01–4.86%), respectively. The proportion of species affected by Cu in the waters of Jiaomen (S3), and Hongqimen (S4) water were slightly higher than that at other sampling sites. For Pb, and Zn, higher mean values of the proportion of species affected were observed in the water of Modaomen (S6) and Hengmen (S5), respectively. The total ecological risks (msPAF) at sampling sites S1, S2, S3, S4, S5, S6, S7, S8 and S9 were 3.87% (0.45–7.52%), 3.55% (1.17–7.19%), 4.37% (2.23–6.75%), 4.91% (2.06–8.51%), 4.19% (1.08–7.52%), 3.52% (0.52–6.73%), 3.87% (0.85–7.32%),3.40% (1.04–6.23%), and 3.02% (0.92–6.42%), respectively. The water of Hongqimen (S4) and Yamen (S9) displayed the highest and lowest mean values of msPAF, respectively.

## Discussion

In recent years, the total concentration of heavy metals in estuarine waters of the Pearl River has declined, but the concentrations of Zn and As have not changed significantly^44^. According to the “China’s Sea Water Quality Standard (GB3097-1997)”, the average level of As in all sampling sites met the Grade I water quality standard (≤ 20 μg/L). The mean Pb content at 9 sampling sites did not match the Grade I (≤ 1 μg/L), but all reached the Grade II standard (≤ 5 μg/L). With the exception of Hongqimen (S4), the mean Cu concentration in other estuaries met the water quality standard of Grade I (≤ 5 μg/L), and the water of Hongqimen reached the water quality standard of Grade II (≤ 10 μg/L). The average Zn concentration in the water of Humen (S2) did not match the water quality standard of Grade I (≤ 20 μg/L) but reached the water quality standard of Grade II (≤ 50 μg/L). The average Zn concentration at other sampling sites met the water quality standard of Grade I. For Hg, the average concentration at all sampling sites did not meet the Grade I standard(≤ 0.05 μg/L), but met the water quality standard of Grade II (≤ 0.2 μg/L) with the exception of Jiaomen (S3). The Hg content in Jiaomen water was slightly higher than the water quality standard of Grade II. In this study, we tested and found that the Cd content in estuarine waters of the Pearl River was very low. The mean Cd contents in the water of Jiaomen (S3), Humen (S2) were 0.10 μg/L, and 0.08 μg/L, respectively, and Cd contents even lower than 0.06 μg/L at other sampling sites. This is the reason why this study did not consider ecological risk of Cd on aquatic organisms. The mean As, Cu, Zn, Cd, Pb and Hg concentrations in the Pearl River estuary are higher than in the Yangtze River estuary^19,45^. The levels of Cu, As and Pb in the waters of the Pearl River estuary is similar to that of Bohai Bay, but the contents of Zn, Cd, and Hg in the waterbodies of Bohai Bay are much higher than those of the Pearl River estuary^5,46^. In general, the heavy metal concentrations in the water of Humen (S2), Jiaomen (S3), Hongqimen (S4), and Hengmen (S5) were slightly higher than the other sampling sites, and the heavy metal content in the water of Yamen (S9) was the lowest. This is mainly due to the fact that the river upstream of Humen, Jiaomen, Hongqimen, and Hengmen passes through the main manufacturing cities of the Pearl River Delta, such as Foshan, Dongguan, ZhongShan and Guangzhou, whereas the river upstream of Yamen passes by Jiangmen, which is less developed in the manufacturing industry. Upstream manufacturing companies can be the major source of heavy metals in the estuarine waters of the Pearl River^44,47^.

High concentrations of heavy metals were detected in aquatic organisms in coastal areas water of South China, and the mollusks had higher levels of heavy metals than other species^1^. Crustacean *Artemia salina* and *Acartia tonsa* were the most susceptible aquatic organism to As (Fig. 3) and Hg (Fig. 7), respectively. However, HC_5_ and RQ values suggest that the ecological risks of As and Hg on aquatic organisms at each sampling site were low (Table 1), and the proportion of affected species induced by As and Hg was below 0.008% and 0.08%, respectively (Fig. 8). The ecological risk induced by As can almost be ignored, but the ecological risk caused by Hg needs some attention although the ecological risk is currently low. Liu et al. reported a significant increase in Hg concentrations in seawater, and a high bioaccumulation of Hg in seafood was observed^6^. The fish *Engraulis japonicus* showed the greatest susceptibility to the heavy metal Pb in the SSD cuvers (Fig. 5), but the fish *Engraulis japonicus* is very secure due to HC_5_ and RQ values indicating very low ecological risk (Table 1). Although Jia et al. and Xiao et al. noted that Pb poses a low ecological risk in estuarine waters of the Pearl River^44,47^, certain hot spots also deserve much more attention for Pb contamination. The sampling sites with the higher risks induced by Pb were Hongqimen (S4), Modaomen (S6) and Hutiaomen (S8) with the proportion of affected species reached 0.57% (0.08-1.55%), 0.56% (<1.29%) and 0.59% (0.01-1.49%), respectively (Fig. 8). The RQ values indicating that there were certain ecological risks caused by Cu and Zn (Table 1). The proportion of species affected by Cu and Zn was relatively high, particularly in the water of Shijiaozui (S1), Humen (S2), Jiaomen (S3), and Hongqimen (S4). The proportion of species affected by Cu was 3.16% (1.64-4.62%) and 3.39% (1.37-4.86%) in the water of Jiaomen (S3) and Hongqimen (S4), respectively. The proportion of species impacted by Zn exceed 1.00% in the water of Shijiaozui (S1), Humen (S2), and Jiaomen (S3) water (Fig. 8). The mollusk *Corbicula fluminea* and the crustacean *Temora stylifera* were the most sensitive aquatic organisms to the heavy metal Cu (Fig. 4) and Zn (Fig. 6), respectively. The Zn content has a lethal effect on the crustaceans *Temora Stylifera* because the LC_50_ of *Temora Stylifera* range from 4μg/L to 90μg/L with average value of 32.875 μg/L (Table S7). Zn was found to be the most dominant heavy metal in fish in Guangdong coastal waters with concentrations of (19.93–67.63) mg kg^−1^ dry weight, and the fish *Coiliamystus* had the highest concentration, followed by *Liza carinatus*^17^. The Cu concentration has a serious impact on the mollusks *Corbicula Fluminea* (the LC_50_ range from 4.2 μg/L to 52.5 μg/L with average value of 22.46 μg/L (TableS8)) and has a certain impact on the crustaceans *Corophium sp.* (the LC_50_ range from 9 μg/L to 99 μg/L with average value of 60.5 μg/L (Table S8)) and the fish *Sparus aurata* (the average value of LC_50_ is 70 μg/L (Table S8)). Mao et al. and Jia et al. also indicated that Cu poses a high ecological risk to aquatic organisms in the estuarine waters of the Pearl River^44,48^. In addition to estuaries of the Pearl River, Cu and Zn have been found to pose a great ecological risk to aquatic organisms in other adjacent seas of China, such as estuaries of the Yangtze River and the Bohai Bay^10^. In the waters of China's coastal shellfish aquaculture areas, Cu and Zn posed higher ecological risks than Cd, Pb, and Hg, and most breeding areas had msPAF values above 20%, indicating highly ecological risks^13^.

Solely from analysis of heavy metal content in water, the content of As, Pb and Hg in the estuarine waters of the Pearl River had little effect on the selected aquatic organisms, but Cu and Zn were hazardous to aquatic organisms. The higher joint ecological risks (msPAF) for five heavy metals were observed at Jiaomen (S3), Hongqimen (S4) and Hengmen (S5) with the proportion of species affected were 4.37% (2.23–6.75%), 4.91% (2.06–8.51%), and 4.19% (1.08–7.52%), respectively (Fig. 8). It must be noted that some toxic heavy metals are very low in the water but very high in the sediment, resulting in a high ecological hazard to benthic aquatic organisms. Zhang et al. studied the heavy metals in sediment from urban river (Panyu and Nansha district) in the upper reaches of Jiaomen (S3), and found that Cd content in sediments was high with moderate ecological risk^3^. The much higher Cd concentration in sediments can be explained by the fact that Cd is mainly enriched in the sediment in the form of reducible fractions (linked to Fe–Mn)^3^. Furthermore, new emerging contaminants in water, including antibiotics, microplastics, pesticides and phenols, will pose ecological risks to aquatic organisms^49–53^; however, heavy metals in sediment and other chemical pollutants in water were not considered in the SSD analysis in this study due to limited data.

Species Sensitivity Distribution (SSD) is a statistical distribution model widely used for ecological risk assessment and water quality baseline. It applies to virtually all chemical pollutants, and the larger the sample, the greater the reliability of the results^54^. Zheng et al. compared sensitivities of aquatic species to heavy metals (Cu, Hg, Cd, Cr^6+^, Pb, Zn) and found that invertebrate taxa was more sensitive than vertebrates to each heavy metal^35^. In this study, the SSD curves (Figs. 3, 4, 5, 6, 7) showed no significant differences in sensitivity between species group. This problem stems from the fact that this study involved too few aquatic organisms to obtain the different distribution. In the case of a large proportion of aquatic organisms in the Pearl River estuary that did not undergo toxicity testing, it is difficult to determine harmful concentration of heavy metals. Toxicological tests for local species should be strengthened to establish a toxicological database on native organisms.

In this study, estuaries with high risk of heavy metal pollution in the Pearl River Estuary were preliminary identified, and aquatic species with high ecological risk were preliminary screened out. Research findings can serve as a basis for formulating water quality standards for heavy metals and for protecting aquatic biodiversity in the Pearl River Estuary. To improve heavy metals pollution in the Pearl River estuary and reduce the ecological risk for aquatic organisms, there is a need to strengthen oversight and control of wastewater sources in upper rivers. It is necessary to strictly monitor releases of wastewater from companies that cause significant pollution, such as printing and dyeing of textiles, metal processing and electronics. Firms should be encouraged to improve their production processes and to reduce the use of toxic and dangerous heavy metals^44^.

## Conclusions

Generally speaking, concentrations of As, Cu, Pb, Hg and Zn in estuarine waters of the Pearl River were not high. With the exception of Hg in Jiaomen water, the other heavy metals in in each sampling site met or exceeded the water quality standard of Grade II. Heavy metal levels and joint ecological risks (msPAF) in the Humen, Jiaomen, Hongqimen, and Hengmen estuaries were slightly higher than in other estuaries, and the Yamen estuary had the lowest contration of heavy metals and ecological risk. The aquatic ecological risks of As, Pb and Hg were generally low in the waters of the Pearl River estuary. Cu and Zn have some effects on aquatic organisms and the proportion of species affected was 3.39% and 1.05% in the Hongqimen and Humen estuaries, respectively. The content of Zn has a lethal effect on the crustaceans *Temora Stylifera*; the content of Cu has a serious impact on the mollusks *Corbicula Fluminea* and has a certain impact on the crustaceans *Corophium sp.* and the fish *Sparus aurata*.

In this study, the combination of RQ values and SSD curves was used to identify the types and regions of pollution by heavy metals and aquatic organisms that pose ecological risks. However, due to data limitations, the risks to aquatic life in the Pearl River Estuary were not fully evaluated. To quickly discover threatened aquatic life and better protect biodiversity, it is necessary to conduct dynamic monitoring of the aquatic environment in the Pearl River estuary and to establish a complete local biotoxicological database.

## Supplementary information

Supplementary Tables.

## Data Availability

All source data analysed are available in the supplementary information.
