# Supplementary material for: Ecological risk assessment of aquatic organisms induced by heavy metals in the estuarine waters of the Pearl River
Source: Sci Rep. 2023 Jun 5;13:9145. doi: 10.1038/s41598-023-35798-x (PMC10241906; doi:10.1038/s41598-023-35798-x)
Supplement: Supplementary file 1 — Supplementary Tables. [file 41598_2023_35798_MOESM1_ESM.pdf]

# Supplementary Material

**Ecological risk assessment of aquatic organisms induced by heavy metals in the estuarine waters of the Pearl River**

Zhihua Tang* • Xinyu Liu • Xiaojun Niu • Hua Yin* • Minru Liu* • Dongqing Zhang • Huafang Guo

10 Tables

**Table S1** As contrations (μg/L) in 9 sampling sites at monthly intervals from January to December in 2020

**Table S2** Cu contrations (μg/L) in 9 sampling sites at monthly intervals from January to December in 2020

**Table S3** Pb contrations (μg/L) in 9 sampling sites at monthly intervals from January to December in 2020

**Table S4** Zn contrations (μg/L) in 9 sampling sites at monthly intervals from January to December in 2020

**Table S5** Hg contrations (μg/L) in 9 sampling sites at monthly intervals from January to December in 2020

**Table S6** EC50 and LC50 values of aquatic organisms to heavy metal Pb from the US EPA ECOTOX database used for generating SSD curves

**Table S7** EC50 and LC50 values of aquatic organisms to heavy metal Zn from the US EPA ECOTOX database used for generating SSD curves

**Table S8** EC50 and LC50 values of aquatic organisms to heavy metal Cu from the US EPA ECOTOX database used for generating SSD curves

**Table S9** EC50 and LC50 values of aquatic organisms to heavy metal As from the US EPA ECOTOX database used for generating SSD curves

**Table S10** EC50 and LC50 values of aquatic organisms to heavy metal Hg from the US EPA ECOTOX database used for generating SSD curves

**Table S1** As contrations (μg/L) in 9 sampling sites at monthly intervals from January to December in 2020

| Sampling site | Position of section | Jan. | Feb. | Mar. | Apr. | May | Jun. | July | Aug. | Sep. | Oct. | Nov. | Dec. |
|---|---|---|---|---|---|---|---|---|---|---|---|---|---|
| S1 | Left | 2.03 | 0.90 | 1.31 | 1.82 | 1.71 | 7.00 | 1.36 | 2.42 | 4.48 | 0.90 | 2.66 | 2.56 |
| | Right | 1.91 | 0.99 | 1.34 | 1.73 | 1.50 | 3.87 | 1.33 | 2.49 | 4.42 | 0.83 | 2.70 | 2.49 |
| S2 | Left | 2.23 | 1.45 | 2.10 | 2.19 | 1.33 | 8.53 | 2.02 | 4.71 | 7.08 | 2.22 | 3.50 | 4.23 |
| | Center | 2.20 | 1.38 | 2.19 | 2.11 | 1.51 | 8.42 | 1.86 | 4.49 | 6.40 | 2.52 | 2.38 | 2.58 |
| | Right | 2.29 | 1.43 | 2.13 | 2.46 | 1.26 | 10.81 | 1.94 | 4.47 | 6.56 | 2.58 | 1.99 | 1.82 |
| S3 | Left | 2.36 | 1.42 | 2.40 | 3.27 | 1.44 | 10.66 | 2.24 | 2.99 | 3.90 | 1.76 | 3.49 | 2.30 |
| | Center | 2.66 | 1.47 | 2.15 | 1.94 | 1.38 | 5.06 | 2.29 | 3.19 | 3.08 | 1.79 | 3.64 | 2.19 |
| | Right | 2.53 | 1.46 | 2.19 | 2.12 | 1.28 | 4.91 | 2.15 | 3.39 | 2.88 | 1.88 | 3.30 | 2.45 |
| S4 | Left | 2.05 | 1.46 | 2.23 | 1.54 | 1.12 | 5.47 | 1.35 | 2.89 | 2.85 | 1.47 | 3.31 | 2.44 |
| | Center | 2.06 | 1.44 | 2.30 | 1.46 | 1.32 | 4.63 | 1.30 | 2.74 | 2.89 | 1.02 | 3.19 | 2.80 |
| | Right | 2.10 | 1.61 | 2.30 | 1.53 | 1.34 | 3.37 | 1.31 | 2.98 | 2.92 | 0.86 | 3.51 | 2.86 |
| S5 | Left | 2.02 | 1.37 | 1.86 | 1.21 | 1.39 | 4.50 | 1.24 | 2.40 | 2.64 | 0.57 | 3.80 | 2.61 |
| | Center | 2.00 | 1.32 | 1.94 | 1.13 | 1.40 | 4.06 | 1.34 | 2.40 | 2.75 | 0.60 | 3.84 | 2.87 |
| | Right | 2.11 | 1.55 | 1.97 | 1.33 | 1.34 | 4.07 | 1.36 | 2.49 | 3.27 | 0.78 | 3.83 | 2.64 |
| S6 | Left | 1.95 | 1.31 | 1.98 | 1.53 | 1.28 | 6.08 | 1.25 | 2.16 | 3.00 | 2.31 | 3.58 | 2.81 |
| | Center | 2.02 | 1.36 | 2.06 | 1.31 | 1.55 | 5.20 | 1.33 | 2.20 | 3.31 | 2.11 | 3.42 | 3.04 |
| | Right | 2.03 | 1.47 | 2.00 | 1.21 | 1.19 | 3.96 | 1.21 | 2.24 | 3.27 | 2.23 | 3.32 | 3.16 |
| S7 | Left | 2.35 | 1.39 | 1.84 | 1.22 | 1.18 | 3.85 | 1.38 | 2.22 | 2.90 | 1.47 | 3.08 | 2.42 |
| | Center | 2.11 | 1.50 | 2.14 | 1.21 | 1.47 | 3.48 | 1.36 | 2.23 | 2.99 | 1.33 | 2.83 | 2.67 |
| | Right | 2.16 | 1.44 | 2.39 | 1.25 | 1.36 | 4.16 | 1.51 | 2.26 | 3.12 | 1.52 | 2.73 | 2.66 |
| S8 | Left | 1.91 | 1.37 | 2.03 | 1.46 | 1.06 | 5.74 | 1.08 | 1.98 | 2.95 | 1.30 | 2.82 | 2.64 |
| | Center | 1.98 | 1.36 | 2.04 | 1.45 | 1.31 | 4.64 | 1.10 | 2.01 | 3.07 | 1.58 | 2.62 | 2.58 |
| | Right | 1.89 | 1.32 | 2.21 | 1.49 | 1.31 | 8.72 | 1.06 | 2.04 | 3.10 | 0.84 | 3.25 | 2.42 |
| S9 | Left | 1.75 | 1.26 | 1.35 | 1.04 | 1.16 | 4.08 | 0.93 | 1.21 | 2.65 | 1.58 | 2.33 | 3.56 |
| | Center | 1.79 | 1.26 | 1.29 | 0.87 | 1.21 | 4.97 | 0.96 | 1.12 | 2.57 | 1.54 | 1.94 | 2.37 |
| | Right | 1.74 | 1.26 | 1.36 | 0.98 | 1.12 | 4.44 | 0.67 | 1.01 | 2.60 | 1.57 | 1.82 | 2.26 |

**Table S2** Cu contrations (μg/L) in 9 sampling sites at monthly intervals from January to December in 2020

| Sampling site | Position of section | Jan. | Feb. | Mar. | Apr. | May | Jun. | July | Aug. | Sep. | Oct. | Nov. | Dec. |
|---|---|---|---|---|---|---|---|---|---|---|---|---|---|
| S1 | Left | 8.56 | 1.53 | 9.11 | 3.68 | 8.37 | 1.76 | 0.52 | 8.56 | 1.53 | 9.11 | 3.68 | 8.37 |
| | Right | 9.16 | 7.79 | 0.96 | 4.16 | 8.79 | 1.45 | 0.18 | 9.16 | 7.79 | 0.96 | 4.16 | 8.79 |
| S2 | Left | 6.05 | 0.46 | 4.17 | 2.00 | 5.31 | 2.10 | 2.43 | 6.05 | 0.46 | 4.17 | 2.00 | 5.31 |
| | Center | 1.67 | 5.77 | 8.50 | 1.67 | 5.84 | 1.61 | 0.14 | 1.67 | 5.77 | 8.50 | 1.67 | 5.84 |
| | Right | 6.95 | 9.91 | 2.76 | 2.16 | 6.14 | 1.70 | 1.99 | 6.95 | 9.91 | 2.76 | 2.16 | 6.14 |
| S3 | Left | 7.26 | 1.43 | 7.84 | 4.76 | 7.59 | 3.40 | 6.34 | 7.26 | 1.43 | 7.84 | 4.76 | 7.59 |
| | Center | 1.52 | 7.65 | 0.48 | 5.03 | 7.43 | 3.62 | 6.02 | 1.52 | 7.65 | 0.48 | 5.03 | 7.43 |
| | Right | 8.42 | 3.24 | 8.44 | 4.75 | 6.71 | 3.71 | 5.34 | 8.42 | 3.24 | 8.44 | 4.75 | 6.71 |
| S4 | Left | 2.86 | 0.25 | 3.36 | 5.15 | 4.35 | 5.21 | 4.97 | 2.86 | 0.25 | 3.36 | 5.15 | 4.35 |
| | Center | 5.15 | 7.25 | 1.79 | 6.14 | 4.32 | 5.16 | 7.14 | 5.15 | 7.25 | 1.79 | 6.14 | 4.32 |
| | Right | 7.81 | 7.46 | 9.33 | 6.88 | 4.42 | 5.34 | 9.29 | 7.81 | 7.46 | 9.33 | 6.88 | 4.42 |
| S5 | Left | 1.57 | 2.17 | 8.40 | 4.65 | 2.71 | 3.11 | 6.86 | 1.57 | 2.17 | 8.40 | 4.65 | 2.71 |
| | Center | 8.21 | 2.17 | 0.64 | 3.13 | 2.90 | 2.67 | 7.52 | 8.21 | 2.17 | 0.64 | 3.13 | 2.90 |
| | Right | 0.19 | 9.30 | 7.45 | 7.78 | 3.15 | 2.57 | 4.19 | 0.19 | 9.30 | 7.45 | 7.78 | 3.15 |
| S6 | Left | 9.00 | 3.69 | 9.93 | 3.61 | 2.20 | 1.48 | 7.63 | 9.00 | 3.69 | 9.93 | 3.61 | 2.20 |
| | Center | 0.03 | 2.31 | 6.39 | 3.28 | 1.73 | 1.75 | 9.30 | 0.03 | 2.31 | 6.39 | 3.28 | 1.73 |
| | Right | 4.45 | 5.15 | 8.91 | 2.47 | 1.81 | 1.93 | 13.58 | 4.45 | 5.15 | 8.91 | 2.47 | 1.81 |
| S7 | Left | 5.03 | 6.22 | 6.70 | 3.64 | 4.05 | 1.98 | 11.00 | 5.03 | 6.22 | 6.70 | 3.64 | 4.05 |
| | Center | 6.24 | 6.82 | 1.45 | 3.59 | 3.93 | 1.91 | 12.95 | 6.24 | 6.82 | 1.45 | 3.59 | 3.93 |
| | Right | 9.11 | 1.10 | 1.37 | 6.48 | 4.49 | 3.66 | 10.76 | 9.11 | 1.10 | 1.37 | 6.48 | 4.49 |
| S8 | Left | 2.99 | 5.14 | 7.44 | 2.09 | 4.02 | 1.37 | 8.45 | 2.99 | 5.14 | 7.44 | 2.09 | 4.02 |
| | Center | 7.47 | 2.50 | 6.34 | 2.00 | 2.28 | 1.23 | 13.15 | 7.47 | 2.50 | 6.34 | 2.00 | 2.28 |
| | Right | 2.71 | 3.55 | 3.69 | 2.04 | 2.32 | 1.95 | 8.83 | 2.71 | 3.55 | 3.69 | 2.04 | 2.32 |
| S9 | Left | 7.00 | 2.25 | 4.41 | 0.88 | 8.99 | 2.26 | 5.92 | 7.00 | 2.25 | 4.41 | 0.88 | 8.99 |
| | Center | 7.26 | 4.74 | 7.80 | 0.52 | 8.98 | 2.25 | 0.23 | 7.26 | 4.74 | 7.80 | 0.52 | 8.98 |
| | Right | 4.45 | 1.52 | 0.57 | 0.76 | 8.61 | 2.27 | 0.16 | 4.45 | 1.52 | 0.57 | 0.76 | 8.61 |

**Table S3** Pb contrations (μg/L) in 9 sampling sites at monthly intervals from January to December in 2020

| Sampling site | Position of section | Jan. | Feb. | Mar. | Apr. | May | Jun. | July | Aug. | Sep. | Oct. | Nov. | Dec. |
|---|---|---|---|---|---|---|---|---|---|---|---|---|---|
| S1 | Left | 2.41 | 1.05 | 2.94 | 6.76 | 0.07 | 0.07 | 1.11 | 1.08 | 3.68 | 9.07 | 0.07 | 0.46 |
| | Right | 0.08 | 6.05 | 1.23 | 6.63 | 0.07 | 1.03 | 0.07 | 0.47 | 7.00 | 9.11 | 0.08 | 0.64 |
| S2 | Left | 7.51 | 1.01 | 9.42 | 2.16 | 2.26 | 0.34 | 0.07 | 0.22 | 4.10 | 4.58 | 3.62 | 2.67 |
| | Center | 4.03 | 4.98 | 4.34 | 0.07 | 0.08 | 0.07 | 0.08 | 1.30 | 1.43 | 4.37 | 1.18 | 1.00 |
| | Right | 1.18 | 1.36 | 2.69 | 2.26 | 0.07 | 1.18 | 0.07 | 0.46 | 3.96 | 1.90 | 0.58 | 4.05 |
| S3 | Left | 1.80 | 3.65 | 2.94 | 5.79 | 0.06 | 0.36 | 0.07 | 0.45 | 6.75 | 1.55 | 0.31 | 0.71 |
| | Center | 1.36 | 1.21 | 8.90 | 9.61 | 0.07 | 0.08 | 0.06 | 0.86 | 1.00 | 3.65 | 0.25 | 0.59 |
| | Right | 1.12 | 7.32 | 8.71 | 8.38 | 0.12 | 1.79 | 0.36 | 0.34 | 8.21 | 4.52 | 0.21 | 0.95 |
| S4 | Left | 9.76 | 0.90 | 5.29 | 6.02 | 0.76 | 0.75 | 1.21 | 0.54 | 0.49 | 0.30 | 0.68 | 1.20 |
| | Center | 7.25 | 6.09 | 7.85 | 6.40 | 1.11 | 0.89 | 1.16 | 0.96 | 0.94 | 4.86 | 0.27 | 5.60 |
| | Right | 8.46 | 10.00 | 6.05 | 6.44 | 1.06 | 0.07 | 0.17 | 0.48 | 2.37 | 3.38 | 0.58 | 1.09 |
| S5 | Left | 9.06 | 4.64 | 1.65 | 1.75 | 0.22 | 0.07 | 1.06 | 0.44 | 3.56 | 3.31 | 0.04 | 1.52 |
| | Center | 0.41 | 5.55 | 2.67 | 0.07 | 1.18 | 0.07 | 1.88 | 0.22 | 2.35 | 3.69 | 0.14 | 1.34 |
| | Right | 7.37 | 7.83 | 6.31 | 3.70 | 1.91 | 0.07 | 1.16 | 2.73 | 3.07 | 2.11 | 0.03 | 1.46 |
| S6 | Left | 7.49 | 8.07 | 5.15 | 4.72 | 8.24 | 0.06 | 1.28 | 0.33 | 4.11 | 0.15 | 0.07 | 0.70 |
| | Center | 4.62 | 2.76 | 7.73 | 2.09 | 5.28 | 0.07 | 2.85 | 5.80 | 2.30 | 2.25 | 0.04 | 0.60 |
| | Right | 8.64 | 1.60 | 1.14 | 7.01 | 1.76 | 0.07 | 0.27 | 1.02 | 3.79 | 2.51 | 0.04 | 1.81 |
| S7 | Left | 0.78 | 1.59 | 1.91 | 7.01 | 0.73 | 0.07 | 9.55 | 0.97 | 5.47 | 4.03 | 0.07 | 0.94 |
| | Center | 1.59 | 7.85 | 5.95 | 4.75 | 3.65 | 0.96 | 0.74 | 1.28 | 1.91 | 5.02 | 0.33 | 0.65 |
| | Right | 0.79 | 6.57 | 2.10 | 5.56 | 0.07 | 0.07 | 0.12 | 0.90 | 0.90 | 3.17 | 0.21 | 0.70 |
| S8 | Left | 7.28 | 8.93 | 3.05 | 1.79 | 0.09 | 0.07 | 0.76 | 0.49 | 5.97 | 5.68 | 0.13 | 0.31 |
| | Center | 8.88 | 3.79 | 4.26 | 0.30 | 4.65 | 0.07 | 0.16 | 0.12 | 8.81 | 4.59 | 0.07 | 0.22 |
| | Right | 5.70 | 3.06 | 8.52 | 2.40 | 4.66 | 0.07 | 0.15 | 1.10 | 9.60 | 8.08 | 0.07 | 0.24 |
| S9 | Left | 0.26 | 1.62 | 9.69 | 2.50 | 0.07 | 0.07 | 0.26 | 0.84 | 2.64 | 7.18 | 0.07 | 1.00 |
| | Center | 8.45 | 8.02 | 1.03 | 0.16 | 1.27 | 1.59 | 0.40 | 1.07 | 3.05 | 1.81 | 0.07 | 2.56 |
| | Right | 5.44 | 4.66 | 0.34 | 1.44 | 0.07 | 0.31 | 0.42 | 1.22 | 2.07 | 5.27 | 0.07 | 0.33 |

**Table S4** Zn contrations (μg/L) in 9 sampling sites at monthly intervals from January to December in 2020

| Sampling site | Position of section | Jan. | Feb. | Mar. | Apr. | May | Jun. | July | Aug. | Sep. | Oct. | Nov. | Dec. |
|---|---|---|---|---|---|---|---|---|---|---|---|---|---|
| S1 | Left | 36.14 | 39.65 | 49.51 | 39.90 | 9.20 | 3.61 | 3.54 | 42.52 | 4.90 | 4.88 | 3.25 | 4.91 |
| | Right | 34.92 | 38.67 | 2.34 | 56.99 | 10.88 | 2.42 | 5.71 | 20.57 | 7.50 | 3.99 | 34.31 | 6.06 |
| S2 | Left | 34.75 | 36.16 | 0.31 | 14.63 | 28.62 | 7.34 | 2.91 | 91.60 | 21.67 | 3.06 | 38.17 | 10.60 |
| | Center | 11.79 | 25.53 | 13.43 | 4.05 | 14.93 | 9.16 | 4.22 | 93.37 | 16.06 | 3.60 | 3.92 | 88.47 |
| | Right | 22.30 | 6.64 | 9.00 | 9.61 | 18.01 | 6.46 | 0.57 | 73.39 | 3.60 | 2.24 | 2.95 | 0.07 |
| S3 | Left | 12.78 | 31.65 | 22.78 | 27.70 | 9.35 | 1.63 | 1.36 | 49.67 | 8.90 | 6.34 | 2.40 | 5.41 |
| | Center | 4.76 | 17.04 | 22.05 | 49.21 | 4.82 | 0.28 | 0.23 | 21.96 | 7.59 | 4.94 | 2.45 | 5.93 |
| | Right | 30.34 | 48.75 | 24.77 | 37.87 | 8.07 | 0.24 | 2.57 | 31.72 | 5.25 | 6.05 | 7.80 | 7.74 |
| S4 | Left | 41.45 | 8.45 | 24.87 | 34.30 | 12.31 | 5.50 | 6.90 | 29.65 | 12.72 | 4.92 | 5.53 | 12.76 |
| | Center | 14.66 | 10.92 | 49.22 | 58.26 | 6.95 | 3.77 | 6.05 | 21.85 | 13.84 | 4.36 | 4.76 | 13.02 |
| | Right | 42.28 | 47.84 | 35.73 | 95.25 | 4.05 | 1.11 | 6.26 | 20.72 | 15.55 | 4.27 | 6.25 | 11.90 |
| S5 | Left | 29.82 | 22.78 | 27.64 | 69.04 | 4.46 | 1.58 | 6.55 | 20.79 | 9.51 | 3.86 | 12.44 | 10.68 |
| | Center | 5.85 | 41.05 | 40.15 | 69.62 | 4.44 | 1.43 | 5.17 | 20.05 | 9.66 | 5.68 | 16.56 | 12.79 |
| | Right | 41.90 | 3.63 | 46.62 | 63.75 | 4.42 | 3.01 | 1.29 | 12.78 | 7.87 | 4.06 | 17.14 | 22.64 |
| S6 | Left | 5.19 | 45.27 | 23.52 | 69.98 | 4.62 | 2.49 | 4.61 | 18.10 | 10.96 | 0.34 | 3.40 | 9.07 |
| | Center | 11.76 | 24.74 | 4.88 | 64.16 | 3.71 | 2.23 | 2.13 | 16.98 | 10.56 | 0.54 | 1.81 | 6.81 |
| | Right | 29.45 | 0.10 | 16.26 | 38.33 | 3.51 | 3.12 | 0.34 | 17.30 | 3.56 | 1.40 | 14.68 | 3.93 |
| S7 | Left | 10.08 | 8.30 | 37.25 | 52.63 | 5.80 | 3.24 | 0.19 | 20.90 | 12.27 | 2.84 | 16.45 | 15.50 |
| | Center | 41.40 | 5.57 | 0.23 | 22.19 | 9.21 | 3.92 | 0.37 | 12.29 | 25.89 | 0.83 | 11.29 | 6.34 |
| | Right | 4.14 | 7.94 | 42.67 | 27.35 | 7.91 | 3.58 | 1.44 | 16.98 | 1.53 | 2.95 | 3.16 | 6.48 |
| S8 | Left | 32.42 | 10.10 | 14.33 | 7.95 | 10.36 | 4.79 | 1.40 | 17.58 | 12.38 | 1.50 | 3.75 | 4.49 |
| | Center | 10.47 | 6.08 | 46.91 | 4.79 | 4.58 | 4.51 | 1.79 | 12.27 | 7.68 | 1.25 | 2.18 | 4.62 |
| | Right | 19.81 | 26.98 | 20.77 | 15.63 | 4.45 | 4.75 | 4.15 | 28.99 | 2.08 | 0.31 | 32.43 | 4.92 |
| S9 | Left | 7.56 | 45.55 | 43.68 | 11.63 | 14.65 | 3.22 | 4.13 | 16.26 | 8.37 | 0.67 | 6.55 | 1.17 |
| | Center | 18.55 | 29.83 | 20.05 | 2.80 | 17.12 | 4.11 | 3.93 | 20.35 | 10.99 | 3.86 | 4.41 | 2.19 |
| | Right | 35.73 | 5.56 | 2.78 | 13.27 | 25.89 | 5.19 | 2.05 | 36.52 | 2.27 | 1.03 | 3.00 | 3.91 |

**Table S5** Hg contrations (μg/L) in 9 sampling sites at monthly intervals from January to December in 2020

| Sampling site | Position of section | Jan. | Feb. | Mar. | Apr. | May | Jun. | July | Aug. | Sep. | Oct. | Nov. | Dec. |
|---|---|---|---|---|---|---|---|---|---|---|---|---|---|
| S1 | Left | 0.13 | 0.15 | 0.14 | 0.07 | 0.18 | 0.10 | 0.16 | 0.13 | 0.05 | 0.09 | 0.10 | 0.10 |
| | Right | 0.16 | 0.12 | 0.11 | 0.10 | 0.18 | 0.13 | 0.04 | 0.17 | 0.07 | 0.11 | 0.18 | 0.13 |
| S2 | Left | 0.01 | 0.19 | 0.16 | 0.08 | 0.13 | 0.14 | 0.20 | 0.20 | 0.20 | 0.02 | 0.20 | 0.05 |
| | Center | 0.21 | 0.04 | 0.20 | 0.04 | 0.07 | 0.16 | 0.16 | 0.20 | 0.11 | 0.07 | 0.02 | 0.09 |
| | Right | 0.18 | 0.13 | 0.06 | 0.18 | 0.15 | 0.15 | 0.15 | 0.16 | 0.07 | 0.02 | 0.10 | 0.22 |
| S3 | Left | 0.22 | 0.20 | 0.42 | 0.18 | 0.32 | 0.25 | 0.40 | 0.43 | 0.28 | 0.37 | 0.19 | 0.02 |
| | Center | 0.19 | 0.04 | 0.50 | 0.33 | 0.08 | 0.33 | 0.18 | 0.06 | 0.19 | 0.10 | 0.05 | 0.41 |
| | Right | 0.50 | 0.31 | 0.11 | 0.01 | 0.38 | 0.46 | 0.17 | 0.16 | 0.21 | 0.03 | 0.18 | 0.22 |
| S4 | Left | 0.19 | 0.21 | 0.02 | 0.06 | 0.06 | 0.10 | 0.12 | 0.15 | 0.14 | 0.09 | 0.02 | 0.21 |
| | Center | 0.20 | 0.15 | 0.13 | 0.12 | 0.05 | 0.15 | 0.22 | 0.22 | 0.12 | 0.22 | 0.11 | 0.21 |
| | Right | 0.15 | 0.16 | 0.08 | 0.20 | 0.16 | 0.11 | 0.05 | 0.17 | 0.05 | 0.19 | 0.11 | 0.09 |
| S5 | Left | 0.10 | 0.05 | 0.08 | 0.10 | 0.02 | 0.12 | 0.11 | 0.13 | 0.09 | 0.08 | 0.11 | 0.07 |
| | Center | 0.06 | 0.07 | 0.07 | 0.03 | 0.10 | 0.14 | 0.05 | 0.12 | 0.12 | 0.01 | 0.05 | 0.12 |
| | Right | 0.10 | 0.02 | 0.14 | 0.01 | 0.05 | 0.10 | 0.09 | 0.08 | 0.08 | 0.04 | 0.02 | 0.04 |
| S6 | Left | 0.09 | 0.12 | 0.10 | 0.01 | 0.12 | 0.04 | 0.02 | 0.10 | 0.03 | 0.06 | 0.07 | 0.04 |
| | Center | 0.12 | 0.06 | 0.07 | 0.04 | 0.10 | 0.04 | 0.02 | 0.09 | 0.05 | 0.06 | 0.04 | 0.07 |
| | Right | 0.05 | 0.02 | 0.01 | 0.09 | 0.08 | 0.11 | 0.01 | 0.11 | 0.10 | 0.10 | 0.01 | 0.06 |
| S7 | Left | 0.08 | 0.13 | 0.12 | 0.11 | 0.10 | 0.04 | 0.08 | 0.10 | 0.04 | 0.05 | 0.13 | 0.05 |
| | Center | 0.01 | 0.01 | 0.03 | 0.07 | 0.10 | 0.01 | 0.10 | 0.07 | 0.02 | 0.11 | 0.06 | 0.13 |
| | Right | 0.11 | 0.13 | 0.10 | 0.13 | 0.12 | 0.12 | 0.11 | 0.13 | 0.10 | 0.13 | 0.13 | 0.04 |
| S8 | Left | 0.22 | 0.02 | 0.24 | 0.20 | 0.02 | 0.17 | 0.19 | 0.07 | 0.19 | 0.12 | 0.05 | 0.16 |
| | Center | 0.08 | 0.14 | 0.19 | 0.15 | 0.01 | 0.01 | 0.19 | 0.19 | 0.04 | 0.10 | 0.10 | 0.16 |
| | Right | 0.11 | 0.07 | 0.14 | 0.11 | 0.18 | 0.01 | 0.16 | 0.10 | 0.10 | 0.11 | 0.04 | 0.11 |
| S9 | Left | 0.06 | 0.06 | 0.08 | 0.08 | 0.06 | 0.07 | 0.05 | 0.07 | 0.06 | 0.04 | 0.08 | 0.03 |
| | Center | 0.05 | 0.04 | 0.07 | 0.03 | 0.09 | 0.04 | 0.04 | 0.05 | 0.05 | 0.07 | 0.08 | 0.03 |
| | Right | 0.04 | 0.07 | 0.06 | 0.04 | 0.09 | 0.04 | 0.05 | 0.09 | 0.07 | 0.04 | 0.09 | 0.04 |

**Table S6** EC50 and LC50 values of aquatic organisms to heavy metal Pb from the US EPA ECOTOX database used for generating SSD curves

| **Pb-crustacena** | **Exposure (μg/L)** | **Pb-fish** | **Exposure (μg/L)** | **Pb-mollusc** | **Exposure (μg/L)** |
|---|---|---|---|---|---|
| Grandidierella japonica | 1560 | Engraulis japonicus | 5 | Meretrix meretrix | 7160 |
| Fenneropenaeus penicillatus | 15800 | Epinephelus sp. | 22500 | Meretrix meretrix | 297 |
| Fenneropenaeus penicillatus | 2000 | Epinephelus sp. | 42500 | Meretrix meretrix | 199 |
| Crangon crangon | 8800 | Epinephelus sp. | 19000 | Meretrix meretrix | 7160 |
| Cancer magister | 600 | Epinephelus sp. | 17000 | Meretrix meretrix | 353 |
| Cancer magister | 575 | Therapon jarbua | 1600 | Corbicula fluminea | 1023320 |
| Artemia sp. | 1700 | Therapon jarbua | 3040 | Mytilus edulis | 3692.6 |
| Artemia sp. | 1400 | Therapon jarbua | 1230 | Cerithidea cingulata | 23972 |
| Acartia clausi | 668 | Therapon jarbua | 2200 | Cerithidea cingulata | 15507 |
| Acartia clausi | 668 | Epinephelus sp. | 22500 | Cerithidea cingulata | 36380 |
| | | Epinephelus sp. | 42500 | Cerithidea cingulata | 64839 |
| | | Epinephelus sp. | 19000 | Babylonia areolata | 12440 |
| | | Epinephelus sp. | 17000 | Babylonia areolata | 22210 |
| | | Engraulis japonicus | 5 | Babylonia areolata | 10500 |
| | | | | Babylonia areolata | 14860 |
| | | | | Argopecten irradians | 8600 |

**Table S7** EC50 and LC50 values of aquatic organisms to heavy metal Zn from the US EPA ECOTOX database used for generating SSD curves

| **Zn-crustacena** | **Exposure (μg/L)** | **Zn-fish** | **Exposure (μg/L)** | **Zn-mollusc** | **Exposure (μg/L)** |
|---|---|---|---|---|---|
| Corophium volutator | 27000 | Engraulis japonicus | 149 | Corbicula fluminea | 2430 |
| Corophium volutator | 7000 | Ambassis sp. | 3900 | Corbicula fluminea | 1580 |
| Corophium volutator | 4400 | Ambassis sp. | 1000 | Corbicula fluminea | 2070 |
| Corophium volutator | 4400 | Ambassis sp. | 8400 | Corbicula fluminea | 6040 |
| Corophium volutator | 128000 | Ambassis sp. | 6600 | Corbicula fluminea | 50 |
| Corophium volutator | 12000 | Ambassis sp. | 4300 | Corbicula fluminea | 590 |
| Corophium volutator | 25000 | Therapon jarbua | 17370 | Corbicula fluminea | 40 |
| Corophium volutator | 15000 | Therapon jarbua | 13700 | Corbicula fluminea | 490 |
| Corophium volutator | 95000 | Therapon jarbua | 19450 | Corbicula fluminea | 13.4 |
| Corophium volutator | 4600 | Therapon jarbua | 17780 | Planaxis sulcatus | 33450 |
| Corophium volutator | 44000 | Therapon jarbua | 11750 | Planaxis sulcatus | 5260 |
| Corophium volutator | 1900 | Therapon jarbua | 8700 | Ostrea sp. | 2000 |
| Corophium volutator | 90000 | Therapon jarbua | 17380 | Ostrea sp. | 632 |
| Corophium volutator | 17000 | Therapon jarbua | 13000 | Nerita albicilla | 6280 |
| Corophium volutator | 11000 | Therapon jarbua | 11000 | Nerita albicilla | 4510 |
| Corophium volutator | 1600 | Therapon jarbua | 15000 | Mya arenaria | 3100 |
| Corophium volutator | 20000 | Therapon jarbua | 19600 | Mya arenaria | 320000 |
| Corophium volutator | 46000 | | | Mya arenaria | 7700 |
| Corophium volutator | 1700 | | | Haliotis rubra | 1730 |
| Corophium volutator | 1100 | | | Haliotis rubra | 4900 |
| Corophium volutator | 1000 | | | | |
| Corophium volutator | 31000 | | | | |
| Corophium volutator | 1700 | | | | |
| Corophium volutator | 128000 | | | | |
| Corophium volutator | 128000 | | | | |
| Corophium volutator | 14000 | | | | |
| Corophium volutator | 12000 | | | | |
| Corophium volutator | 2700 | | | | |
| Corophium volutator | 128000 | | | | |
| Corophium volutator | 16000 | | | | |
| Corophium volutator | 65000 | | | | |
| Corophium volutator | 110000 | | | | |
| Corophium volutator | 3400 | | | | |
| Corophium volutator | 16000 | | | | |
| Corophium volutator | 128000 | | | | |
| Corophium volutator | 1000 | | | | |
| Corophium volutator | 100000 | | | | |

| **Zn-crustacena** | **Exposure (μg/L)** | **Zn-fish** | **Exposure (μg/L)** | **Zn-mollusc** | **Exposure (μg/L)** |
|---|---|---|---|---|---|
| Corophium volutator | 8500 | | | | |
| Corophium volutator | 13000 | | | | |
| Corophium volutator | 128000 | | | | |
| Corophium volutator | 128000 | | | | |
| Corophium volutator | 128000 | | | | |
| Corophium volutator | 90000 | | | | |
| Corophium volutator | 3000 | | | | |
| Corophium volutator | 6500 | | | | |
| Corophium volutator | 3600 | | | | |
| Corophium volutator | 46000 | | | | |
| Corophium volutator | 54000 | | | | |
| Corophium volutator | 1100 | | | | |
| Corophium volutator | 128000 | | | | |
| Corophium volutator | 17000 | | | | |
| Corophium volutator | 3200 | | | | |
| Corophium volutator | 14120 | | | | |
| Corophium volutator | 9790 | | | | |
| Palaemonetes pugio | 11300 | | | | |
| Palaemon northropi | 16000 | | | | |
| Temora stylifera | 30 | | | | |
| Temora stylifera | 4 | | | | |
| Temora stylifera | 14 | | | | |
| Temora stylifera | 31 | | | | |
| Temora stylifera | 23 | | | | |
| Temora stylifera | 31 | | | | |
| Temora stylifera | 40 | | | | |
| Temora stylifera | 90 | | | | |
| Talitrus saltator | 62740 | | | | |
| Scylla serrata | 489800 | | | | |
| Scylla serrata | 398100 | | | | |
| Scylla serrata | 32000 | | | | |
| Scylla serrata | 645700 | | | | |
| Scylla serrata | 741300 | | | | |
| Scutellidiumsp. | 1090 | | | | |
| Penaeus chinensis | 870 | | | | |
| Penaeus chinensis | 48 | | | | |
| Penaeus chinensis | 645 | | | | |
| Penaeus chinensis | 87 | | | | |
| Pagurus longicarpus | 200 | | | | |
| Pagurus longicarpus | 400 | | | | |
| Pagurus longicarpus | 12000 | | | | |
| Nitocra spinipes | 1300 | | | | |

| **Zn-crustacena** | **Exposure (μg/L)** | **Zn-fish** | **Exposure (μg/L)** | **Zn-mollusc** | **Exposure (μg/L)** |
|---|---|---|---|---|---|
| Nitocra spinipes | 1450 | | | | |
| Nitocra spinipes | 890 | | | | |
| Nitocra spinipes | 2800 | | | | |
| Nitocra spinipes | 4300 | | | | |
| Nitocra spinipes | 2400 | | | | |
| Fenneropenaeus penicillatus | 1370 | | | | |
| Fenneropenaeus penicillatus | 7850 | | | | |
| Fenneropenaeus penicillatus | 1340 | | | | |
| Fenneropenaeus penicillatus | 3580 | | | | |
| Fenneropenaeus penicillatus | 2660 | | | | |
| Fenneropenaeus penicillatus | 1430 | | | | |
| Fenneropenaeus penicillatus | 1820 | | | | |
| Fenneropenaeus penicillatus | 1610 | | | | |
| Fenneropenaeus penicillatus | 2170 | | | | |
| Fenneropenaeus penicillatus | 1210 | | | | |
| Fenneropenaeus penicillatus | 4980 | | | | |
| Fenneropenaeus penicillatus | 50 | | | | |
| Fenneropenaeus penicillatus | 1310 | | | | |
| Fenneropenaeus penicillatus | 2380 | | | | |
| Fenneropenaeus penicillatus | 1690 | | | | |
| Fenneropenaeus penicillatus | 2970 | | | | |
| Fenneropenaeus penicillatus | 100 | | | | |
| Fenneropenaeus penicillatus | 1370 | | | | |

| Zn-crustacena | Exposure (μg/L) | Zn-fish | Exposure (μg/L) | Zn-mollusc | Exposure (μg/L) |
|---|---|---|---|---|---|
| Fenneropenaeus penicillatus | 7850 | | | | |
| Fenneropenaeus penicillatus | 1340 | | | | |
| Fenneropenaeus penicillatus | 3580 | | | | |
| Fenneropenaeus penicillatus | 2660 | | | | |
| Fenneropenaeus penicillatus | 1430 | | | | |
| Fenneropenaeus penicillatus | 1820 | | | | |
| Fenneropenaeus penicillatus | 1610 | | | | |
| Fenneropenaeus penicillatus | 2170 | | | | |
| Fenneropenaeus penicillatus | 1210 | | | | |
| Fenneropenaeus penicillatus | 4980 | | | | |
| Fenneropenaeus penicillatus | 50 | | | | |
| Fenneropenaeus penicillatus | 1310 | | | | |
| Fenneropenaeus penicillatus | 2380 | | | | |
| Fenneropenaeus penicillatus | 1690 | | | | |
| Fenneropenaeus penicillatus | 2970 | | | | |
| Fenneropenaeus penicillatus | 100 | | | | |
| Fenneropenaeus penicillatus | 3500 | | | | |
| Fenneropenaeus penicillatus | 3500 | | | | |
| Carcinus maenas | 1000 | | | | |
| Carcinus maenas | 14500 | | | | |
| Carcinus maenas | 14870 | | | | |

**Table S8** EC50 and LC50 values of aquatic organisms to heavy metal Cu from the US EPA ECOTOX database used for generating SSD curves

| Cu-crustacena | Exposure (μg/L) | Cu-fish | Exposure (μg/L) | Cu-mollusc | Exposure (μg/L) |
|---|---|---|---|---|---|
| Corophium sp. | 99 | Engraulis mordax | 185 | Corbicula fluminea | 4.2 |
| Corophium sp. | 28.5 | Engraulis mordax | 391 | Corbicula fluminea | 40.9 |
| Corophium sp. | 86 | Engraulis mordax | 457 | Corbicula fluminea | 10.9 |
| Corophium sp. | 9 | Engraulis mordax | 199 | Corbicula fluminea | 21.4 |
| Corophium sp. | 80 | Engraulis mordax | 374 | Corbicula fluminea | 52.5 |
| Corophium volutator | 20740 | Engraulis mordax | 412 | Corbicula fluminea | 8.1 |
| Corophium volutator | 37590 | Engraulis mordax | 523 | Corbicula fluminea | 19.2 |
| Grandidierella japonica | 250 | Engraulis mordax | 398 | | |
| Palaemonetes pugio | 37000 | Engraulis mordax | 485 | | |
| Palaemonetes pugio | 37000 | Engraulis mordax | 235 | | |
| Palaemonetes pugio | 2150 | Engraulis mordax | 213 | | |
| Palaemonetes pugio | 2560 | Engraulis mordax | 409 | | |
| Palaemonetes pugio | 1230 | Engraulis mordax | 292 | | |
| Palaemonetes pugio | 1410 | Engraulis mordax | 193 | | |
| Palaemonetes pugio | 1690 | Engraulis mordax | 375 | | |
| Palaemonetes pugio | 750 | Engraulis mordax | 186 | | |
| Palaemonetes pugio | 127.1 | Engraulis mordax | 391 | | |
| Palaemonetes pugio | 317 | Engraulis mordax | 372 | | |
| Palaemonetes pugio | 508 | Engraulis mordax | 409 | | |
| Palaemonetes pugio | 2100 | Engraulis mordax | 186 | | |
| Palaemon northropi | 16000 | Engraulis mordax | 457 | | |
| | | Engraulis mordax | 412 | | |
| | | Engraulis mordax | 199 | | |
| | | Engraulis mordax | 374 | | |
| | | Engraulis mordax | 213 | | |
| | | Engraulis mordax | 292 | | |
| | | Engraulis mordax | 370 | | |
| | | Engraulis mordax | 185 | | |
| | | Engraulis mordax | 193 | | |
| | | Engraulis mordax | 485 | | |
| | | Engraulis mordax | 398 | | |
| | | Engraulis mordax | 186 | | |
| | | Engraulis mordax | 523 | | |
| | | Engraulis mordax | 235 | | |
| | | Engraulis mordax | 375 | | |
| | | Ambassis sp. | 190 | | |
| | | Ambassis sp. | 3100 | | |
| | | Ambassis sp. | 1300 | | |

| **Cu-crustacena** | **Exposure (μg/L)** | **Cu-fish** | **Exposure (μg/L)** | **Cu-mollusc** | **Exposure (μg/L)** |
|---|---|---|---|---|---|
| | | Ambassis sp. | 1200 | | |
| | | Ambassis sp. | 1500 | | |
| | | Ambassis sp. | 2000 | | |
| | | Ambassis sp. | 3800 | | |
| | | Ambassis sp. | 1100 | | |
| | | Ambassis sp. | 1400 | | |
| | | Ambassis sp. | 1100 | | |
| | | Ambassis sp. | 1400 | | |
| | | Ambassis sp. | 1500 | | |
| | | Coryphaena hippurus | 15.3 | | |
| | | Coryphaena hippurus | 32.8 | | |
| | | Sparus sp. | 1170 | | |
| | | Sparus sp. | 1280 | | |
| | | Sparus sp. | 1340 | | |
| | | Sparus aurata | 64 | | |
| | | Sparus aurata | 76 | | |
| | | Therapon jarbua | 2240 | | |
| | | Therapon jarbua | 4260 | | |
| | | Therapon jarbua | 3160 | | |
| | | Therapon jarbua | 2570 | | |
| | | Therapon jarbua | 3230 | | |
| | | Therapon jarbua | 6100 | | |
| | | Therapon jarbua | 4500 | | |
| | | Therapon jarbua | 9650 | | |
| | | Therapon jarbua | 6650 | | |

**Table S9** EC50 and LC50 values of aquatic organisms to heavy metal As from the US EPA ECOTOX database used for generating SSD curves

| **As-crustacena** | **Exposure (μg/L)** | **As-fish** | **Exposure (μg/L)** | **As-mollusc** | **Exposure (μg/L)** |
|---|---|---|---|---|---|
| Corophium volutator | 36000 | Therapon jarbua | 4080 | Corbicula fluminea | 600 |
| Corophium volutator | 60000 | Therapon jarbua | 3680 | Corbicula fluminea | 1380 |
| Corophium volutator | 128000 | Therapon jarbua | 5500 | Corbicula fluminea | 350 |
| Corophium volutator | 5600 | Therapon jarbua | 3380 | Corbicula fluminea | 380 |
| Corophium volutator | 12000 | saxatilis Morone | 10300 | Corbicula fluminea | 3480 |
| Corophium volutator | 6000 | Oreochromis mossambicus | 26500 | Corbicula fluminea | 4650 |
| Corophium volutator | 7500 | Oncorhynch us keta | 11000 | | |
| Corophium volutator | 11000 | Morone saxatilis | 18960 | | |
| Corophium volutator | 17000 | Danio rerio | 272000 | | |
| Corophium volutator | 2200 | | | | |
| Corophium volutator | 58000 | | | | |
| Corophium volutator | 23000 | | | | |
| Corophium volutator | 16000 | | | | |
| Corophium volutator | 14000 | | | | |
| Corophium volutator | 128000 | | | | |
| Corophium volutator | 7500 | | | | |
| Corophium volutator | 22000 | | | | |
| Corophium volutator | 2500 | | | | |
| Corophium volutator | 120000 | | | | |
| Corophium volutator | 3090 | | | | |
| Corophium volutator | 11000 | | | | |
| Corophium volutator | 20000 | | | | |
| Corophium volutator | 1800 | | | | |
| Corophium volutator | 6000 | | | | |
| Corophium volutator | 23000 | | | | |
| Corophium volutator | 20000 | | | | |
| Corophium volutator | 15000 | | | | |
| Corophium volutator | 42000 | | | | |
| Corophium volutator | 7000 | | | | |
| Corophium volutator | 5800 | | | | |
| Corophium volutator | 3000 | | | | |
| Corophium volutator | 6000 | | | | |
| Corophium volutator | 14000 | | | | |
| Corophium volutator | 16000 | | | | |
| Corophium volutator | 42000 | | | | |
| Corophium volutator | 54000 | | | | |
| Corophium volutator | 40000 | | | | |

| **As-crustacena** | **Exposure (μg/L)** | **As-fish** | **Exposure (μg/L)** | **As-mollusc** | **Exposure (μg/L)** |
|---|---|---|---|---|---|
| Corophium volutator | 2600 | | | | |
| Corophium volutator | 128000 | | | | |
| Corophium volutator | 50000 | | | | |
| Corophium volutator | 44000 | | | | |
| Corophium volutator | 2200 | | | | |
| Corophium volutator | 5400 | | | | |
| Corophium volutator | 1800 | | | | |
| Corophium volutator | 5600 | | | | |
| Corophium volutator | 14000 | | | | |
| Tigriopus japonicus | 17200 | | | | |
| Nitocra spinipes | 3000 | | | | |
| Crangon crangon | 47000 | | | | |
| Crangon crangon | 96000 | | | | |
| Crangon crangon | 70000 | | | | |
| Artemia salina | 257 | | | | |
| Ampelisca abdita | 4160 | | | | |
| Ampelisca abdita | 5110 | | | | |
| Ampelisca abdita | 8460 | | | | |
| Ampelisca abdita | 8000 | | | | |

**Table S10** EC50 and LC50 values of aquatic organisms to heavy metal Hg from the US EPA ECOTOX database used for generating SSD curves

| **Hg-crustacena** | **Exposure (μg/L)** | **Hg-fish** | **Exposure (μg/L)** | **Hg-mollusc** | **Exposure (μg/L)** |
|---|---|---|---|---|---|
| Petrolisthes armatus | 64 | Therapon jarbua | 71 | Meretrix meretrix | 234.6 |
| Petrolisthes armatus | 50 | Therapon jarbua | 60 | Meretrix meretrix | 13.3 |
| Petrolisthes armatus | 54 | Therapon jarbua | 71 | Meretrix meretrix | 5.4 |
| Petrolisthes armatus | 60 | Leiostomus xanthurus | 36 | Meretrix meretrix | 14 |
| Petrolisthes armatus | 64 | Leiostomus xanthurus | 36 | Meretrix meretrix | 109.3 |
| Petrolisthes armatus | 65 | calcarifer Lates | 85 | Perna viridis | 280 |
| Petrolisthes armatus | 64 | calcarifer Lates | 200 | Perna viridis | 560 |
| Penaeus monodon | 18 | calcarifer Lates | 112.8 | Perna viridis | 155 |
| Penaeus merguiensis | 30 | | | Perna viridis | 135 |
| Penaeus merguiensis | 70 | | | Perna viridis | 230 |
| Penaeus merguiensis | 30 | | | Perna viridis | 270 |
| Penaeus merguiensis | 160 | | | Perna viridis | 295 |
| Penaeus merguiensis | 290 | | | Perna viridis | 250 |
| Penaeus merguiensis | 130 | | | Perna viridis | 1000 |
| Palaemonetes vulgaris | 400 | | | Perna viridis | 202 |
| Palaemonetes pugio | 90 | | | Perna viridis | 225 |
| Palaemonetes pugio | 200 | | | Perna viridis | 520 |
| Palaemonetes pugio | 2600 | | | Perna viridis | 155 |
| Palaemon serratus | 3368 | | | Perna viridis | 230 |
| Nitocra spinipes | 230 | | | Perna viridis | 125 |
| Macrobrachium rosenbergii | 380 | | | Perna viridis | 160 |
| Macrobrachium rosenbergii | 440 | | | Ostrea sp. | 10 |
| Macrobrachium rosenbergii | 580 | | | Ostrea sp. | 32 |
| Macrobrachium rosenbergii | 175 | | | Nassarius obsoletus | 32000 |
| Macrobrachium rosenbergii | 368 | | | Nassarius obsoletus | 700 |
| Macrobrachium rosenbergii | 450 | | | Nassarius obsoletus | 32000 |
| Macrobrachium rosenbergii | 210 | | | Crepidula fornicata | 25000 |
| Macrobrachium rosenbergii | 70 | | | Crepidula fornicata | 330 |

| Hg-crustacena | Exposure (μg/L) | Hg-fish | Exposure (μg/L) | Hg-mollusc | Exposure (μg/L) |
|---|---|---|---|---|---|
| Macrobrachium rosenbergii | 105 | | | Crepidula fornicata | 1100 |
| Macrobrachium rosenbergii | 425 | | | Crepidula fornicata | 60 |
| Macrobrachium rosenbergii | 725 | | | | |
| Macrobrachium rosenbergii | 368 | | | | |
| Macrobrachium rosenbergii | 215 | | | | |
| Macrobrachium rosenbergii | 165 | | | | |
| Macrobrachium rosenbergii | 310 | | | | |
| Macrobrachium rosenbergii | 430 | | | | |
| Macrobrachium rosenbergii | 500 | | | | |
| Macrobrachium rosenbergii | 125 | | | | |
| Macrobrachium rosenbergii | 592 | | | | |
| Macrobrachium rosenbergii | 400 | | | | |
| Macrobrachium rosenbergii | 215 | | | | |
| Macrobrachium rosenbergii | 400 | | | | |
| Macrobrachium rosenbergii | 155 | | | | |
| Macrobrachium rosenbergii | 85 | | | | |
| Macrobrachium rosenbergii | 910 | | | | |
| Macrobrachium rosenbergii | 340 | | | | |
| Macrobrachium rosenbergii | 150 | | | | |
| Macrobrachium rosenbergii | 145 | | | | |
| Macrobrachium rosenbergii | 115 | | | | |

| Hg-crustacena | Exposure (μg/L) | Hg-fish | Exposure (μg/L) | Hg-mollusc | Exposure (μg/L) |
|---|---|---|---|---|---|
| Macrobrachium rosenbergii | 415 | | | | |
| Macrobrachium rosenbergii | 425 | | | | |
| Macrobrachium rosenbergii | 50 | | | | |
| Macrobrachium rosenbergii | 330 | | | | |
| Macrobrachium rosenbergii | 450 | | | | |
| Macrobrachium rosenbergii | 390 | | | | |
| Macrobrachium rosenbergii | 510 | | | | |
| Macrobrachium rosenbergii | 500 | | | | |
| Macrobrachium rosenbergii | 745 | | | | |
| Macrobrachium rosenbergii | 235 | | | | |
| Macrobrachium rosenbergii | 565 | | | | |
| Macrobrachium rosenbergii | 125 | | | | |
| Macrobrachium rosenbergii | 445 | | | | |
| Macrobrachium rosenbergii | 800 | | | | |
| Macrobrachium rosenbergii | 940 | | | | |
| Macrobrachium rosenbergii | 400 | | | | |
| Macrobrachium rosenbergii | 340 | | | | |
| Macrobrachium rosenbergii | 475 | | | | |
| Macrobrachium rosenbergii | 800 | | | | |
| Macrobrachium rosenbergii | 365 | | | | |
| Macrobrachium rosenbergii | 415 | | | | |

| **Hg-crustacena** | **Exposure (μg/L)** | **Hg-fish** | **Exposure (μg/L)** | **Hg-mollusc** | **Exposure (μg/L)** |
|---|---|---|---|---|---|
| Macrobrachium rosenbergii | 1260 | | | | |
| Fenneropenaeus penicillatus | 6 | | | | |
| Fenneropenaeus penicillatus | 2 | | | | |
| Fenneropenaeus penicillatus | 11.3 | | | | |
| Fenneropenaeus penicillatus | 154 | | | | |
| Fenneropenaeus penicillatus | 45 | | | | |
| Fenneropenaeus penicillatus | 22.99 | | | | |
| Fenneropenaeus penicillatus | 9.03 | | | | |
| Fenneropenaeus penicillatus | 191 | | | | |
| Fenneropenaeus penicillatus | 84 | | | | |
| Fenneropenaeus penicillatus | 55 | | | | |
| Fenneropenaeus penicillatus | 54 | | | | |
| Fenneropenaeus penicillatus | 4.09 | | | | |
| Fenneropenaeus penicillatus | 4.06 | | | | |
| Fenneropenaeus penicillatus | 127 | | | | |
| Fenneropenaeus penicillatus | 53.83 | | | | |
| Fenneropenaeus penicillatus | 15.9 | | | | |
| Fenneropenaeus penicillatus | 56 | | | | |
| Fenneropenaeus penicillatus | 111 | | | | |
| Fenneropenaeus penicillatus | 111 | | | | |
| Fenneropenaeus penicillatus | 6.71 | | | | |

| **Hg-crustacena** | **Exposure (μg/L)** | **Hg-fish** | **Exposure (μg/L)** | **Hg-mollusc** | **Exposure (μg/L)** |
|---|---|---|---|---|---|
| Carcinus maenas | 1200 | | | | |
| Carcinus maenas | 1200 | | | | |
| Carcinus maenas | 14 | | | | |
| Carcinus maenas | 8.2 | | | | |
| Carcinus maenas | 6.6 | | | | |
| Carcinus maenas | 21.1 | | | | |
| Acartia tonsa | 15 | | | | |
| Acartia tonsa | 19 | | | | |
| Acartia tonsa | 10 | | | | |
| Acartia tonsa | 16 | | | | |
| Acartia tonsa | 17.5 | | | | |
| Acartia tonsa | 13 | | | | |
| Acartia tonsa | 22 | | | | |
| Acartia tonsa | 19 | | | | |
| Acartia tonsa | 17 | | | | |
| Acartia tonsa | 20 | | | | |
| Acartia tonsa | 20 | | | | |
| Acartia tonsa | 14 | | | | |
| Acartia tonsa | 17 | | | | |
| Acartia tonsa | 34 | | | | |
| Acartia tonsa | 3 | | | | |
